# Unveiling the metabolic profile and anti-inflammatory potential of apple of Sodom (*Calotropis procera* (Aiton) W.T.) by UPLC–MS/MS chemometric analysis

**DOI:** 10.1038/s41598-025-33542-1

**Published:** 2026-02-06

**Authors:** Alaa A. El-Banna, Reham S. Ibrahim, Doaa A. Ghareeb, Sarah M. Nassief

**Affiliations:** 1 Department of Pharmacognosy, Faculty of Pharmacy, Alexandria University, Alkhartoom Square, Alexandria, 21521 Egypt; 2 Bio-Screening and Preclinical Trial Lab, Biochemistry Department, Faculty of Science, Alexandria University, Alexandria, Egypt; 3 Center of Excellence for Drug Preclinical Studies (CE-DPS), Pharmaceutical and Fermentation Industry Development Center, City of Scientific Research and Technological Applications (SRTA-City), New Borg El-Arab City, Alexandria, Egypt; 4 Research Projects Unit, Pharos University, Alexandria, Egypt

**Keywords:** *Calotropis procera*, Inflammation, UPLC–MS/MS analysis, Multivariate analysis, Drug discovery, Biomarkers

## Abstract

*Calotropis procera*, commonly referred to as the apple of Sodom, is a perennial shrub, notable for its ecological role and extensive use in traditional medicine. This study aims to explore the phytochemical variability among different organs’ extracts of *C. procera*, specifically stems, leaves, flowers, fruits, and seeds, using ultra-performance liquid chromatography-tandem mass spectrometry (UPLC–MS/MS) and multivariate statistical techniques. A comprehensive analysis identified 112 distinct phytoconstituents, with unique profiles observed across the various organs’ extracts. The study further assessed the anti-inflammatory potential of these extracts by examining their effects on the gene expression of key pro-inflammatory cytokines (TNF-α, IL-1β, IL-6, and INF-γ) in lipopolysaccharide (LPS)-stimulated human white blood cells. Results indicate that the extracts from flowers and fruits significantly downregulated IL-1β expression to levels lower than those exhibited by piroxicam, indicating their proficient inflammation inhibitory efficacy. Orthogonal Projection to Latent Structures (OPLS) coefficient plots unraveled that methyldimethoxycinnamate, vinyloxybutanol, butanediol dibenzoate, pyroterebic acid and ursenone are the key biomarkers accountable for this efficacy. This research underscores *C. procera*'s pharmacological significance, providing a scientific foundation for its traditional medicinal applications. The findings highlight the necessity for further investigations into the mechanistic pathways of its bioactive compounds and their implications for developing novel anti-inflammatory therapies.

**Supplementary Information:**

The online version contains supplementary material available at 10.1038/s41598-025-33542-1.

## Introduction

*Calotropis procera* (Aiton) W.T. is a perennial shrub belonging to the family Apocynaceae, subfamily Asclepiadaceae, and it is indigenous to Africa and the Arabian Peninsula. It is commonly known as apple of Sodom^1,2^.

This plant has garnered attention not only for its ecological significance but also for its extensive use in traditional medicine. Ecologically, *C. procera* plays a vital role in its native habitats, contributing to soil stabilization and preventing erosion due to its extensive root system^3^. Whereas traditionally, various parts of *C. procera*, including leaves, stems, and roots, have been employed in folk remedies to treat a myriad of ailments, particularly those associated with inflammation, gastrointestinal discomfort, fevers and infections, highlighting the plant’s versatility in traditional healing practices^4–6^.

*Calotropis procera* has been found to contain a wide range of phytochemicals, including alkaloids, flavonoids, tannins, saponins, terpenoids, and glycosides. The specific compounds and their concentrations can vary depending on the plant part (leaf, root, flower, latex). It also contains important compounds like linoleic acid, squalene, and cardiac glycosides, as well as various phenolic acids and fatty acid esters. These compounds are responsible for many of the plant’s traditional medicinal uses^7,8^.

Inflammation is a complex biological response to harmful stimuli, playing a crucial role in the body’s defense mechanism^9^. The underlying mechanisms of inflammation involve various cellular and molecular processes, including the release of pro-inflammatory cytokines, activation of immune cells, and the production of reactive oxygen species^9^. The search for effective anti-inflammatory agents has led researchers to explore natural products, with plant extracts emerging as promising candidates due to their diverse phytochemical profiles and relatively low side effects compared to synthetic drugs^10^.

Previous studies have indicated that *C. procera* possesses significant pharmacological properties, including anti-inflammatory, analgesic, and antioxidant effects^11^. For instance, the latex of *C. procera* has been shown to exhibit potent anti-inflammatory activity against various mediators of inflammation, such as cyclooxygenase and lipoxygenase enzymes, making it a subject of interest in pharmacological research^12^. Various compounds, such as flavonoids, alkaloids, saponins, and tannins, have been isolated from different organs of the plant, each contributing to its therapeutic potential^13^. These compounds are known to modulate inflammatory pathways, reducing the production of pro-inflammatory mediators and enhancing the body’s antioxidant defenses^11^.

Despite the promising findings, comprehensive studies examining the anti-inflammatory activity of extracts from different organs of *C. procera* remain limited. Therefore, this research aims to investigate the phytochemical variability among *C. procera* different organs (stems, leaves, flowers, fruits, and seeds) using UPLC–MS/MS technique along with multivariate statistical analysis to pinpoint the qualitative and quantitative phytochemical markers responsible for such variability. After that, the assessment of the anti-inflammatory potentials of these different organs’ extracts was conducted by studying their impacts on the gene expression of some pro-inflammatory cytokines (TNF-*α*, IL-6, IL-1*β*, INF-*γ*) by applying polymerase chain reaction (PCR). Through the construction of orthogonal projections to latent structures (OPLS) coefficient plots, the exact phytoconstituents accountable for these anti-inflammatory potentials were specified in each organ’s extract. This study seeks to elucidate the potential of *C. procera* as a natural anti-inflammatory agent. The findings may provide a scientific basis for the traditional uses of this plant and contribute to the development of new therapeutic strategies for managing inflammatory conditions, thereby enhancing our understanding of its pharmacological potential and paving the way for future research.

## Experimental

### Plant collection

In July 2024, five *C. procera* samples were collected during the flowering-fruiting stage from Najran town in Saudi Arabia. The plant was collected according to the national guidelines. The samples were authenticated by Prof. Dr. Salama El Dareer, Professor of Botany, Faculty of Science, Alexandria University. A voucher specimen (CP 2024) was preserved at the Department of Pharmacognosy, Faculty of Pharmacy, Alexandria University.

### Sample preparation

Each *C. procera* sample was separated into five organs: stems, seeds, flowers, fruits, and leaves. Then the separated organs were left to dry at room temperature. After that, 50 g of each dried organ was ground into powder using an electric grinder and extracted with 100 ml of 70% ethanol through ultra-sonication at 50 °C for 30 min. Five extracts of each organ were prepared, then filtered, concentrated under reduced pressure until dry. The total number of prepared extracts was 25. This extraction method was applied for its simplicity, energy efficiency, and its ability to provide good extraction of the metabolites.

### Chemical profiling of *C. procera* extracts using UPLC–MS/MS

#### Preparation of extracts for UPLC–MS analysis

The dry extracts were prepared to a concentration of 1 mg/mL using HPLC-grade methanol and filtered using a 0.2 µm membrane disc filter. Sonication was applied to degas the samples before injection. A volume of 10 µL of each sample was injected in full loop mode into the chromatographic column, with each sample analysed five times.

#### UPLC experimental conditions

The secondary metabolites in the *C. procera* extracts were analysed using an UPLC XEVO TQD triple quadrupole mass spectrometer (Waters Corporation, Milford, MA, USA). The system included a Waters Acquity QSM pump, LC-2040 autosampler, degasser, and a Waters Acquity CM detector. A Waters Acquity UPLC BEH C18 column (50 mm length, 2.1 mm internal diameter, 1.7 µm particle size) was used for chromatographic separation at a flow rate of 0.2 mL/min and a column temperature of 30 °C. The mobile phase consisted of two components: phase A, ultrapure water with 0.1% formic acid, and phase B, methanol with 0.1% formic acid. This composition ensured effective compounds separation. The gradient elution program was as follows: 0.0–2.0 min, 10% eluent B; 2.0–5.0 min, 30% eluent B; 5.0–15.0 min, 70% eluent B; 15.0–22.0 min, 90% eluent B; 22.0–25.0 min, 90% eluent B; 26.0 min, 100% eluent B; 26.0–29.0 min, 100% eluent B; 30.0–40 min, 10% eluent B, followed by 4 min for column re-equilibration.

#### Electrospray Ionization-mass spectrometry (ESI–MS) conditions and metabolites identification

The extracts were analysed in both negative and positive ionization modes. The triple quadrupole mass spectrometer with electrospray ionization (ESI) was used to identify metabolites. The mass analyser employed tandem MS methods, with the first and third quadrupoles acting as mass filters and the second quadrupole as a collision cell for fragmenting parent ions using collision-induced dissociation (CID) in nitrogen gas. The ESI source settings were: 3 kV capillary voltage, 35 V cone voltage, ion source temperature at 150 °C, nebulizer pressure at 35 psi, and drying/sheath gas temperature at 440 °C and 350 °C, respectively. Nitrogen gas flows were set to 900 L/h for drying and 50 L/h for sheath gas. The total analysis time was 30 min, with a full range acquisition covering 50–1000 *m/z*. MS/MS fragmentation was performed with collision energies ranging from 30 to 70 eV for the parent ions. Daughter ions generated during fragmentation were monitored to infer the molecular structure of the parent ions. Metabolite identification was based on retention times, MS/MS fragmentation patterns, and comparison with an in-house database, literature data, and the CRC phytochemical dictionary.

### Anti-inflammatory activity assessment of different *C. procera* organs’ extracts

Cytotoxicity was evaluated for the various extracts in comparison with piroxicam using the MTT (3-(4,5-dimethylthiazol-2-yl)-2,5-diphenyltetrazolium bromide) assay, which is a colorimetric assay used to assess cell metabolic activity, and is often used in cell viability and cytotoxicity studies. The effective anti-inflammatory concentrations (EAICs) of each extract were determined in lipopolysaccharide (LPS)-stimulated human white blood cell cultures. IL-1β, IL-6, TNF-α, and INF-γ expression levels were quantified using real-time polymerase chain reaction (PCR). Results were expressed as means of three independent replicates. Detailed procedural information can be found in the supplementary file.

### Statistical analysis

Multivariate statistical analysis of metabolic data obtained from UPLC–ESI–MS/MS, along with biological results, was carried out using the SIMCA-P Ver 14.0 software (Umetrics, Malmö, Sweden). SIMCA is Soft Independent Modeling of Class Analogy, which is a software used for multivariate data analysis. Unsupervised pattern recognition, specifically principal component analysis (PCA), was applied to identify sample clustering. Additionally, supervised discriminant analysis methods, such as orthogonal partial least squares discriminant analysis (OPLS-DA), were used for samples classification and the identification of distinguishing chemical compounds. Furthermore, an orthogonal partial least squares (OPLS) model was used to identify key biologically relevant markers linked to anti-inflammatory potential, based on the coefficient plots. Hierarchical clustering analysis-heat map (HCA-heat map) was constructed using Metaboanalyst 6.0 (https://www.metaboanalyst.ca/MetaboAnalyst/ModuleView.xhtml).

## Results and discussion

### The use of UPLC–MS/MS for characterization of phytoconstituents in different *C. procera* organs’ extracts

**Fig. 1:**
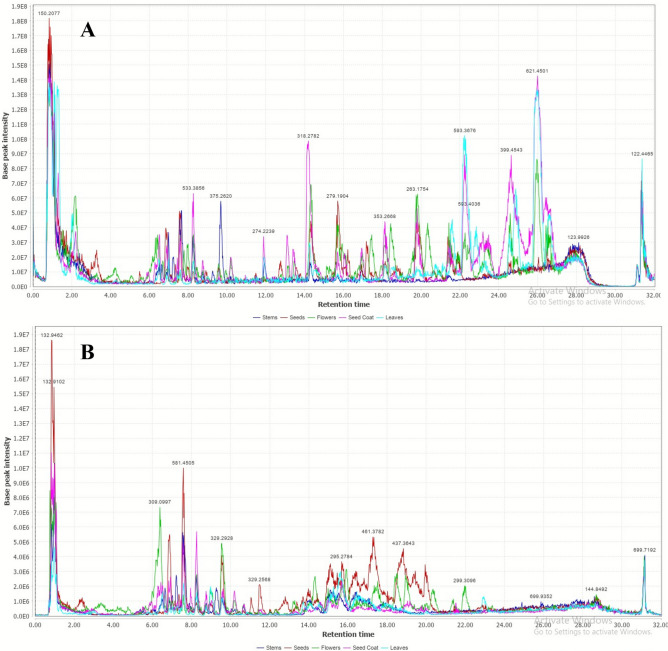
The base peak chromatograms of the extracts of different *C. procera* organs in positive (**A**) and negative (**B**) ion modes.

The UPLC–MS/MS technique enabled the tentative identification of 112 phytoconstituents in *C. procera* different organs’ extracts (Fig. 1 and Table 1). This identification was achieved by comparing the retention times of the compounds to those of external standards and analyzing their MS spectra in conjunction with previously published data, MassBank, and the Natural Products Dictionary database. The identified compounds were categorized into several chemical classes, including butanediol derivatives, dihydroxycinnamic acid derivatives, benzenediol derivatives, pentenoic acid derivatives, flavonoids, cardenolides, terpenoids, and fatty acids.

**Table 1:** The phytoconstituents annotated in the extracts of *C. procera* different organs via UPLC–MS/MS in negative and positive ion modes.

Peak No.	Rt (min.)	Identified compounds	Ion type	Molecular weight	Chemical class	Element composition	MSn fragments
1	0.94	Butanediol diglucuronoside	[M + H]^+^, 443.38	442.37	Butanediol derivative	C_16_H_26_O_14_	195.15, 91.13, 73.06, 55.09
2	2.88	Methoxymelilotoside	[M + H]^+^, 357.23	356.32	Dihydroxy cinnamic acid derivative	C_16_H_20_O_9_	326.2, 313.22, 195.09, 162.14,135, 107, 92.9, 31.03
3	3.21	Methoxymelilotoside-Me ester	[M + H]^+^, 371.3	370.35	Dihydroxy cinnamic acid derivative	C_17_H_22_O_9_	356.27, 340.27, 327.29, 209.16, 135, 107, 92.9
4	5.50	Methoxy phenol	[M + H]^+^, 125.8	124.14	Benzenediol derivative	C_7_H_8_O_2_	95, 81, 53, 39
5	5.76	Dihydroxycinnamic acid	[M − H]^−^, 179.03	180.16	Dihydroxy cinnamic acid derivative	C_9_H_8_O_4_	135.02, 107.01, 92.98
6	6.33	Dimethoxybenzene	[M + H]^+^, 139.0	138.16	Benzenediol derivative	C_8_H_10_O_2_	109, 82, 81, 53, 39
7	6.46	Dihydroxycinnamic acid-Me ether	[M − H]^−^, 193.05	194.18	Dihydroxy cinnamic acid derivative	C_10_H_10_O_4_	179.02, 135.01, 107, 92.97
8	6.70	Diethoxybenzene	[M − H]^−^, 165.0	166.22	Benzenediol derivative	C_17_H_22_O_9_	107, 82, 81, 53, 39
9	6.9	Dihydroxycinnamic acid-di-Me ether, Me ester	[M + H]^+^, 223.24	222.24	Dihydroxy cinnamic acid derivative	C_12_H_14_O_4_	181.15, 137.14, 109.13, 95.1
10	7.0	Dihydroxycinnamic acid-di-Me ether, Et ester	[M + H]^+^, 237.18	236.26	Dihydroxy cinnamic acid derivative	C_13_H_16_O_4_	209.12, 181.07, 137.06, 109.05, 95.02
11	7.78	Dihydroxy-dimethoxyflavone-O-[deoxyhexosyl-hexoside]	[M + H]^+^, 623.5	622.6	Flavonoid	C_29_H_34_O_15_	461, 315, 287, 300, 285
12	7.88	Pentahydroxy-methoxyflavone-O-Sulfate,O- hexoside	[M + H]^+^, 575.6	574.5	Flavonoid	C_22_H_22_O_16_S	413, 333, 318, 305
13	7.91	Pentahydroxy-methoxyflavone-O-Glucuronopyranoside	[M + H]^+^, 509.4	508.4	Flavonoid	C_22_H_20_O_14_	333, 318, 305
14	8.06	Pentahydroxy-methoxyflavone-O-Xyloside	[M − H]^−^, 463.4	464.4	Flavonoid	C_21_H_20_O_12_	331, 316, 303
15	8.24	Pentahydroxy-methoxyflavone-O-deoxyhexoside	[M + H]^+^, 479.2	478.4	Flavonoid	C_22_H_22_O_12_	333, 318, 305
16	8.34	Acetylpatulitrin	[M + H]^+^, 537.4	536.4	Flavonoid	C_24_H_24_O_14_	333, 318, 305
17	8.46	Isobutyrylpatulitrin	[M − H]^−^, 563.4	564.5	Flavonoid	C_26_H_28_O_14_	331, 316, 303
18	8.70	Methylbutyryl patulitrin	[M − H]^−^, 577.4	578.5	Flavonoid	C_27_H_30_O_14_	331, 316, 303
19	8.91	Patuletin feruloylhexoside	[M − H]^−^, 669.4	670.6	Flavonoid	C_32_H_30_O_16_	331, 316, 303
20	9.25	Butanediol, dibenzoate	[M − H]^−^, 297.19	298.33	Butanediol derivative	C_18_H_18_O_4_	121.11, 77.10, 73.06, 55.05
21	9.31	Butanediol, monobenzoate	[M + H]^+^, 195.03	194.23	Butanediol derivative	C_11_H_14_O_3_	121.11, 77.10, 73.06, 56.1
22	9.54	Methoxybutanol	[M + H]^+^, 105.12	104.15	Butanediol derivative	C_5_H_12_O_2_	87.1, 74.09, 55.05, 31.03
23	9.68	Pyroterebic acid	[M + H]^+^, 115.96	114.14	Pentenoic acid derivative	C_6_H_10_O_2_	71.95, 55.10, 43.09
24	9.92	Pyroterebic acid- Amide	[M + H]^+^, 114.55	113.16	Pentenoic acid derivative	C_6_H_11_NO	97.52, 69.51, 55.10, 43.09
25	10.03	Hydroxybutyl acrylate	[M + H]^+^, 145.98	144.17	Butanediol derivative	C_7_H_12_O_3_	127.96, 74.93, 71.05, 55.05
26	10.19	Vinyloxybutanol	[M + H]^+^, 117.97	116.16	Butanediol derivative	C_6_H_12_O_2_	99.95, 74.93, 55.05, 43.04
27	10.20	Dimethoxybutane	[M + H]^+^, 119.77	118.17	Butanediol derivative	C_6_H_14_O_2_	88.74, 73.06, 57.71, 31.03
28	10.32	Butanediol diacrylate	[M − H]^−^, 197.76	198.22	Butanediol derivative	C_10_H_14_O_4_	126.71, 71.05, 55.66
29	10.48	Bis(vinyloxy)butane	[M + H]^+^, 143.99	142.2	Butanediol derivative	C_8_H_14_O_2_	100.95, 73.06, 57.91, 43.04
30	10.77	Butanediol dimethacrylate	[M − H]^−^, 225.14	226.27	Butanediol derivative	C_12_H_18_O_4_	140.06, 73.06, 85.08, 54.98
31	11.47	Heptyloxybutanol	[M − H]^−^, 187.08	188.31	Butanediol derivative	C_11_H_24_O_2_	169.06, 115.19, 71.89, 55.05
32	12.57	Pentahydroxy-methoxyflavone-O-Sulfate	[M + H]^+^, 413.4	412.3	Flavonoid	C_16_H_12_O_11_S	333, 318, 305
33	12.75	Patuletin	[M − H]^−^, 331.3	332.3	Flavonoid	C_16_H_12_O_8_	316, 303
34	13.09	Kumatekillin	[M − H]^−^, 313.3	314.3	Flavonoid	C_17_H_14_O_6_	285, 298, 283
35	13.28	Pentahydroxy-methoxyflavone-Penta-Ac	[M − H]^−^, 541.5	542.4	Flavonoid	C_26_H_22_O_13_	499, 457, 415, 373, 331,316, 303
36	13.36	Dihydroxy-dimethoxyflavone-O-[Hydroxymethyl-methyl-propenyl]	[M + H]^+^, 399.3	398.4	Flavonoid	C_22_H_22_O_7_	315, 300, 285, 287
37	13.41	Dihydroxy-dimethoxyflavone-O-(Hydroxy-methoxyphenyl) ether	[M − H]^−^, 435.4	436.4	Flavonoid	C_24_H_20_O_8_	313, 298, 283, 285
38	13.76	Dihydroxy-oxocard-enolide-carboxylic acid, O-hexoside	[M − H]^−^, 565.4	566.6	Cardenolide	C_29_H_42_O_11_	403,385, 367
39	13.84	Frugosidal	[M + H]^+^, 551.7	550.6	Cardenolide	C_29_H_42_O_10_	405, 387, 369, 351
40	14.25	Digitoxigenin xyloside	[M − H]^−^, 505.4	506.6	Cardenolide	C_28_H_42_O_8_	373, 355, 337
41	14.41	Malayoside	[M + H]^+^, 535.4	534.6	Cardenolide	C_29_H_42_O_9_	389, 371, 353
42	14.55	Coronillobioside	[M − H]^−^, 711.5	712.8	Cardenolide	C_35_H_52_O_15_	549, 387, 369, 351
43	14.84	Boistroside	[M + H]^+^, 519.4	518.6	Cardenolide	C_29_H_42_O_8_	389, 371, 353
44	15.00	Hydroxycardadienolide-O-hexoside	[M + H]^+^, 519.4	518.6	Cardenolide	C_29_H_42_O_8_	357, 339
45	15.04	Digoxigenin digitoxoside	[M + H]^+^, 521.4	520.7	Cardenolide	C_29_H_44_O_8_	391, 373, 355, 337
46	15.12	Christyoside	[M + H]^+^, 549.4	548.7	Cardenolide	C_30_H_44_O_9_	389, 371, 353
47	15.20	Peruvoside	[M + H]^+^, 549.4	548.7	Cardenolide	C_30_H_44_O_9_	389, 371, 353
48	15.33	Proceraside A	[M − H]^−^, 575.4	576.7	Cardenolide	C_31_H_44_O_10_	387, 369, 351
49	15.48	Dihydroxycardenolide-O-[ Xylopyranosyl -allopyranoside]	[M − H]^−^, 667.5	668.8	Cardenolide	C_34_H_52_O_13_	505, 373, 355, 337
50	15.54	Dihydroxy-oxocardenolide-O-[ hexosyl-deoxyhexoside]	[M − H]^−^, 695.4	696.8	Cardenolide	C_35_H_52_O_14_	549, 387, 369, 351
51	15.64	Urezin	[M + H]^+^, 699.5	698.8	Cardenolide	C_35_H_54_O_14_	537, 375, 357, 339
52	15.91	Allopaulioside	[M + H]^+^, 533.6	532.7	Cardenolide	C_30_H_44_O_8_	389, 371, 353
53	16.28	Reevesioside J	[M + H]^+^, 533.6	532.7	Cardenolide	C_30_H_44_O_8_	375, 357, 339
54	16.45	Dihydroxycardenolide-O-[Xylopyranosyl-rhamnopyranoside]	[M + H]^+^, 653.4	652.8	Cardenolide	C_34_H_52_O_12_	507, 375, 357, 339
55	16.70	Digigrandifloroside	[M − H]^−^, 681.5	682.8	Cardenolide	C_35_H_54_O_13_	551, 389, 371, 353, 335
56	16.76	Evobioside	[M + H]^+^, 683.5	682.8	Cardenolide	C_35_H54O_13_	537, 375, 357, 339
57	16.87	Ramnodigin	[M + H]^+^, 489.4	488.7	Cardenolide	C_29_H_44_O_6_	375, 357, 339
58	16.96	Uzarigenin canarobioside	[M + H]^+^, 667.5	666.8	Cardenolide	C_35_H_54_O_12_	537, 375, 357, 339
59	17.28	Oxystelmoside	[M + H]^+^, 667.5	666.8	Cardenolide	C_35_H_54_O_12_	507, 375, 357, 339
60	17.4	Digoxigenin bisdigitoxoside	[M + H]^+^, 651.5	650.8	Cardenolide	C_35_H_54_O_11_	521, 391, 373, 355, 337
61	17.80	Digitoxigenin bisdigitoxide	[M + H]^+^, 635.4	634.8	Cardenolide	C_35_H_54_O_10_	505, 375, 357, 339
62	13.67	Proceralabdanoside	[M + H]^+^, 545.54	544.59	Diterpenoid glycoside	C_26_H_40_O_12_	498, 452, 401, 364, 349, 180, 143
63	18.05	Oleanolic acid-O-[O-Methyl- glycuronoside]	[M + H]^+^, 647.55	646.85	Oleanane-type triterpenoid	C_37_H_58_O_9_	457.7, 442.67¸439, 411, 393, 190.15
64	18.15	Oleanolic acid -O-(O-Acetyl pentose)	[M + H]^+^, 631.66	630.85	Oleanane-type triterpenoid	C_37_H_58_O_8_	457.7, 442.67, 439, 411, 393, 174.15
65	18.25	Anchusoside 1	[M + H]^+^, 781.66	780.98	Oleanane-type triterpenoid	C_42_H_68_O_13_	457.7, 442.67, 439, 411, 393, 162.14
66	18.43	Hederoside C	[M + H]^+^, 735.54	734.96	Oleanane-type triterpenoid	C_41_H_66_O_11_	457.7, 442.67, 439, 411, 393, 278.26
67	18.49	Hydroxylabdanolide-dioic acid	[M + H]^+^, 383.36	382.45	Diterpenoid	C_20_H_30_O_7_	336.42, 290.39, 147.27, 143.12
68	18.57	Dihydroxycardenolide-O-Sulfate	[M − H]^−^, 453.4	454.6	Cardenolide	C_23_H_34_O_7_S	373, 355, 337
69	18.70	Calotropagenin	[M − H]^−^, 403.4	404.5	Cardenolide	C_23_H_32_O_6_	385, 367, 349, 339
70	18.80	Epoxy-trihydroxycardenolide	[M + H]^+^, 405.3	404.5	Cardenolide	C_23_H_32_O_6_	387,369, 351
71	18.95	corotoxigenin	[M + H]^+^, 389.3	388.5	Cardenolide	C_23_H_32_O_5_	371, 353, 343
72	19.05	Digoxigenin	[M + H]^+^, 391.3	390.5	Cardenolide	C_23_H_34_O_5_	373, 355, 337
73	19.31	Epoxy-trihydroxycardenolide-Me ether	[M − H]^−^, 417.4	418.5	Cardenolide	C_24_H_34_O_6_	399, 381
74	19.67	Digitoxigenin	[M + H]^+^, 375.3	374.5	Cardenolide	C_23_H_34_O_4_	357, 339
75	19.69	Dihydroxycardenolide	[M + H]^+^, 375.3	374.5	Cardenolide	C_23_H_34_O_4_	357, 339
76	19.74	Dehydrocalatoxin	[M + H]^+^, 546.6	547.4	Cardenolide glycoside	C_29_H_38_O_10_	385, 367, 349, 339
77	19.84	Anhydroepidigitoxigenin	[M + H]^+^, 357.3	356.5	Cardenolide	C_23_H_32_O_3_	339
78	19.90	Dihydroxycardenolide-Ac	[M − H]^−^, 415.4	416.6	Cardenolide	C_25_H_36_O_5_	372, 354, 336
79	20.10	Trihydroxycardenolide-Di-Ac	[M − H]^−^, 473.4	474.6	Cardenolide	C_27_H_38_O_7_	430,387, 369, 351, 333
80	20.34	Uscharidin	[M + H]^+^, 531.4	530.6	Cardenolide glycoside	C_29_H_38_O_9_	387, 369, 351, 341, 323
81	20.80	Acetoxy-hydroxy-norasclepin	[M − H]^−^, 620.7	619.3	Cardenolide glycoside	C_22_H_44_O_12_	577, 389, 371, 353, 335
82	21.01	Deoxyuscharin	[M − H]^−^, 572.7	573.41	Cardenolide	C_31_H_43_NO_7_S	371, 353, 335
83	21.10	Digitoxigenin monooctadioate	[M − H]^−^, 529.5	530.7	Cardenolide	C_31_H_46_O_7_	373,355, 337
84	21.33	Hydroperoxystigmastenone	[M + H]^+^, 445.28	444.69	Stigmastane derivative	C29H48O3	417.27, 400.26, 303
85	21.43	Hydroxystigmastenone	[M + H]^+^, 429.30	428.69	Stigmastane derivative	C29H48O2	411.28, 401.29, 287.02, 269
86	21.91	Calotroprocerone A	[M + H]^+^, 421.44	420.67	Ursane-type triterpene	C30H44O	231, 218, 150, 98, 70, 121, 82, 67, 54, 28.01
87	22.35	Calotroprocerol A	[M + H]^+^, 423.36	422.69	Ursane-type triterpene	C30H46O	257, 233, 220, 152, 136, 121, 82, 67, 54, 18.02
88	22.46	Taraxadienol	[M + H]^+^, 425.38	424.7	Ursane-type triterpene	C30H48O	235, 222, 154, 136, 121, 82, 67, 54, 18.02
89	22.51	Oleanenone	[M + H]^+^, 425.38	424.7	Oleanene-type triterpenoid	C30H48O	410.35, 382.3, 203.99, 175.98
90	22.73	Ursenone	[M + H]^+^, 425.38	424.7	Ursene-type triterpene	C30H48O	410.22, 382.34
91	23.02	Oleanenol	[M + H]^+^, 427.36	426.72	Oleanene-type triterpenoid	C30H50O	412.33, 394.31
92	23.17	Calotropeol	[M + H]^+^, 427.37	426.72	Ursene-type triterpene	C30H50O	412.34, 409.35
93	23.31	Methoxy ursene	[M + H]^+^, 441.32	440.74	Ursene-type triterpene	C31H52O	426.29, 410.29
94	23.46	Calotropursenyl acetate	[M − H]^−^, 465.27	466.746	Ursene-type triterpene	C32H50O2	450.24, 406.23
95	23.55	Procerursenyl acetate	[M − H]^−^, 465.23	466.75	Ursane-type triterpene	C32H50O2	406, 250, 216, 201, 190, 189, 186, 174, 159
96	24.52	Amyrin propanoate	[M − H]^−^, 481.38	482.78	Ursene-type triterpene	C33H54O2	466.35, 408.31, 73.07
97	24.62	Amyrin butyrate	[M + H]^+^, 497.31	496.81	Ursene-type triterpene	C34H56O2	482.28, 410.21, 87.10
98	24.94	Amyrin pentanoate	[M − H]^−^, 509.35	510.83	Ursene-type triterpene	C35H58O2	494.32, 408.23, 101.12
99	25.23	Amyrin caproate	[M − H]^−^, 523.45	524.86	Ursene-type triterpene	C36H60O2	508.42, 408.3, 115.15
100	25.48	Amyrin caprylate	[M − H]^−^, 551.5	552.91	Ursene-type triterpene	C38H64O2	536.47, 408.3, 143.20
101	26.11	Amyrin hydroxy cinnamate	[M − H]^−^, 571.45	572.86	Ursene-type triterpene	C39H56O3	556.42, 407.29, 164.16
102	26.33	Amyrin cinnamate	[M + H]^+^, 557.47	556.86	Ursene-type triterpene	C39H56O2	542.44, 410.32, 147.15
103	26.57	Amyrin undecanoate	[M + H]^+^, 595.46	594.99	Ursene-type triterpene	C41H70O2	580.43, 410.18, 185.28
104	26.6	Amyrin dodecanoate	[M + H]^+^, 610.47	609.02	Ursene-type triterpene	C42H72O2	595.44, 411.16, 199.31
105	27.16	Amyrin tetradecanoate	[M + H]^+^, 638.44	637.07	Ursene-type triterpene	C44H76O2	623.41, 411.08, 227.36
106	27.3	Amyrin heptadecanoate	[M + H]^+^, 680.48	679.15	Ursene-type triterpene	C47H82O2	665.45, 410.03, 270.45
107	27.37	Hexadecenoic acid	[M + H]^+^, 255.15	254.41	Unsaturated fatty acid	C16H30O2	237.13, 211.14, 179.09, 165.06, 151.03
108	27.51	Methyl hexadecenoate	[M + H]^+^, 269.11	268.43	Unsaturated fatty acid ester	C17H32O2	238.08, 210.07, 196.04, 182.01, 167.98, 31.03
109	27.63	Ethyl hexadecenoate	[M + H]^+^, 283.22	282.46	Unsaturated fatty acid ester	C18H34O2	238.16, 210.15, 196.12, 182.09, 168.06, 45.06
110	27.73	Tetradecyl hexadecenoate	[M − H]^−^, 449.39	450.78	Unsaturated fatty acid ester	C30H58O2	236.01, 208, 193.97, 179.94, 165.91, 213.38
111	28.35	Triacontanol	[M + H]^+^, 439.27	438.82	Fatty alcohol	C30H62O	421.25, 407.22, 393.19, 379.16
112	30.05	Dotriacontene	[M − H]^−^, 447.45	448.86	Alkene	C32H64	433.42, 419.39, 405.36

#### Butanediol derivatives

Compounds **1**, **20–22**, **25–31** were characterized as butanediol derivatives. They all shared fragments ions in their MS2 spectra at *m/z* 73.06 and 55.05 corresponding to [CH_2_=CHCH_2_CH_2_OH + H]^+^ and [CH≡CCH_2_CH_3_ + H]^+^ ions, respectively^14^. Additional different peaks appeared in the mass spectra of these compounds depending on the present substituents. In case of butanediol diglucuronoside (compound **1**) with [M + H]^+^ ion at *m/z* 443.38, an extra peak was observed at *m/z* 195.15 corresponding to loss of glucuronoside moieties^15^. Compounds **20** and **21** showed a daughter peak at *m/z* 121.11 due to benzoate ions removal. These benzoate ions then lost CO_2_ to give phenyl groups represented by a peak at *m/z* 77.10^16^. Compound **20** was with [M − H]^−^ ion at *m/z* 297.19, while compound **21** was with [M + H]^+^ ion at *m/z* 195.03, suggesting that compound **20** is butanediol, dibenzoate. Whereas compound **21** is butanediol, monobenzoate. Compounds **22** and **27** exhibited a peak in their MS2 spectra at *m/z* 31.03 owing to methoxy groups’ loss. Therefore, compound **22** with [M + H]^+^ at *m/z* 105.12 was annotated as methoxybutanol while compound **27** with [M + H]^+^ at *m/z* 119.77 was annotated as dimethoxybutane^17^. For compounds **25** and **28**, a daughter peak was found at *m/z* 71.05 due to removal of acrylate group. Thus, compound **25** with [M + H]^+^ at *m/z* 145.98 was suggested to be hydroxybutyl acrylate, while compound **28** with [M − H]^−^ at *m/z* 197.76 was proposed to be butanediol diacrylate^18^. Compounds **26** and **29** with [M + H]^+^ at *m/z* 117.97 and 143.99 were recognized as vinyloxybutanol and bis(vinyloxy)butane, respectively, as they shared a fragment ion in their MS2 spectra at *m/z* 43.04 corresponding to a vinyloxy group^19^. Compound **30** with [M − H]^−^ at *m/z* 225.14 was identified as butanediol dimethacrylate. It demonstrated daughter peaks at *m/z* 85.08, 140.06, and 54.98 which corresponded to a methacrylate group, and the ions due to sequential loss of the two methacrylate groups, respectively^20^. Compound **31** with [M − H]^−^ at *m/z* 187.08 was supposed to be heptyloxybutanol as it had a daughter peak at *m/z* 115.19 corresponding to a heptyl group^21^.

#### Dihydroxycinnamic acid derivatives

Compounds **2**, **3**, **5**, **7**, **9**, and **10** were recognized as dihydroxycinnamic acid derivatives. They exhibited the same fragmentation pattern which involved the loss of CO_2_ to generate a fragment ion at *m/z* 135, which then subjected to sequential loss of CO and CH_2_ groups to give fragment ions at *m/z* 107 and 92.9, respectively^22^.

Extra peaks were observed in the MS2 spectra of these compounds based on the existing substituents. For methoxymelilotoside (Compound **2**), additional peaks were found at *m/z* 162.14 and 31.03 representing glucosyl and methoxy groups, respectively^23^. Compound **3** shared the same fragmentation pattern of compound **2** with an extra 14.03 Da indicating that it is the methyl ester of methoxymelilotoside (compound **2**). Compound **5** is a typical dihydroxycinnamic acid. Compound **7** that is a methyl ether of compound **5** (with an extra 14.03 Da) showed the same fragment ions with an additional ion [M–H–CH_2_]^−^ at *m/z* 179.02 due to the methyl group loss^22^. Moreover, compounds **9** and **10** demonstrated the same fragment ions of compound **5** with an extra peak at *m/z* 181.15 representing [M–H–3CH_2_]^−^ ion for compound **9**, and additional peaks at *m/z* 209.12 and 181.07 owing to [M–H–2CH_2_]^−^ and [M–H–2CH_2_–C_2_H_4_]^−^ ions, respectively, for compound **10**. Therefore, compounds **9** and **10** were annotated as dihydroxycinnamic acid-di-Me ether, Me ester and dihydroxycinnamic acid-di-Me ether, Et ester, respectively^22^.

#### Benzenediol derivatives

Peaks **4**, **6**, and **8** were identified as methoxy phenol, dimethoxybenzene, and diethoxybenzene, respectively. For peak **4**, the protonated ion peak was observed at 125.8 Da, with daughter ions appearing at 95 Da due to the loss of a methoxy group. Additionally, peaks at *m/z* 82, 81, 69, 53, and 39 might be due to C_6_H_10_, C_6_H_9_^+^, C_5_H_9_^+^, C_4_H_5_^+^ and C_3_H_3_^+^ ions, respectively^24^. Peak **6** exhibited a protonated ion peak at 139.0 Da, accompanied by daughter ions at 109 Da resulting from the loss of two methyl groups. Similarly, peaks at *m/z* 82, 81, 69, 53, and 39 were observed as well. In the case of peak **8**, a deprotonated ion peak was detected at 165.0 Da, with daughter ions observed at 107 Da representing the loss of two ethoxy groups. Similar fragment ions were observed at *m/z* 82, 81, 69, 53, and 39, aligning with the patterns seen for peaks **4** and **6**.

#### Pentenoic acid derivatives

Compound **23** with [M + H]^+^ at *m/z* 115.96 showed daughter peaks in its MS2 spectrum at *m/z* 71.95, 56.10, and 44.09. These peaks represented [M + H-CO_2_]^+^, [CH_2_ = C(CH_3_)_2_]^+^, and [CH_2_(CH_3_)_2_]^+^ ions, respectively. Therefore, compound **23** was suggested to be pyroterebic acid^25^. Whereas compound **24** was found to be the amide derivative of compound **23**. It showed the same fragment ions of compound **24** with extra peaks at *m/z* 97.52 and 69.51 due to sequential removal of amide and carbonyl groups^25^.

#### Flavonoids

This class is represented by 15 peaks, including 9 flavanol glycosides (peaks **11–19**) and 6 aglycones (peaks **32**–**37**). Among the flavonol glycosides, one peak represents a secondary glycoside (peak **11**), while the other 8 peaks are primary glycosides, including five flavonol 3-O glycosides (peaks **12**–**15** and **19**) and three 7-O glycosides (peaks **16**–**18**). In flavonoid glycosides, the size and structure of the sugar portion can be identified in MS^2^ experiments by observing characteristic MS^2^ fragments such as [M–H-162]^−^, ([M–H-146]^−^, and [M–H-132]^−^ which correspond to the loss of O-hexose, O-deoxy hexose, and O-pentose sugars, respectively^26^. The details of the fragmentation patterns of *C. procera*’s identified flavonoids are illustrated in the supplementary file.

#### Cardenolides

Cardenolides are represented by a total of 36 peaks, with 12 peaks attributed to cardenolide aglycones and 24 peaks representing cardenolide glycosides. Cardenolide glycosides are a group of metabolites characterized by an aglycone, which is structurally related to steroid hormones, featuring an unsaturated conjugated lactone ring connected to the C17 position, coupled with one or more sugar moiety^27^. The fragmentation of cardenolides involves the sequential loss of sugar units, accompanied by stepwise elimination of hydroxyl groups from the steroid aglycone moieties, which serves to distinguish between different aglycones^28^. The identified cardenolides of *C. procera* are illustrated with their fragmentation patterns in the supplementary file.

#### Terpenoids

##### Diterpenes

The [M + H]^+^ ion of compound **62** at *m/z* 545.54 is in agreement with the elemental composition of diterpenic glycosides, C_26_H_41_O_12_. It demonstrated fragment ions at *m/z* 498 and 452 which were corresponding to [M–HCOOH]^+^ and [498-HCOOH]^+^ ions, respectively, indicating the existence of two carboxylic moieties. It possessed other daughter peaks at *m/z* 143 and 401 which were due to [C_5_H_6_O_2_COOH]^+^ and [M-143]^+^ ions, respectively. These ions suggested that one carboxylic moiety is in the δ-lactone ring and the other one is in the bicyclic ring system. Other fragment ions appeared at *m/z* 364, 349, and 180 due to [M-C_6_H_12_O_6_]^+^, [364-Me]^+^, and [C_6_H_12_O_6_]^+^ that proved the existence of a hexose moiety in compound **62**. Therefore, compound **62** was annotated as proceralabdanoside^29^. Compound **67** with [M + H]^+^ ion at *m/z* 383.36 is the aglycone of proceralabdanoside (compound **62**). It exhibited the same fragmentation pattern of proceralabdanoside with 162 Da lower due to absence of the hexose moiety^30^.

##### Oleanane-type triterpenes

Six compounds (**63**–**66**, **89**, **91**) were recognized as oleanane-type triterpenes. Four compounds of them (**63**–**66**) were found to be oleanolic acid glycosides. They exhibited fragment ions at *m/z* 190.15, 174.15, 162.14, 278.26, respectively, due to their glycoside moieties. Then the four compounds (**63**–**66**) demonstrated the same fragment ions at *m/z* 457.7, 442.67, 439, 411, and 393 which represented [oleanolic acid + H]^+^, [oleanolic acid + H–CH_3_]^+^, [oleanolic acid + H–H_2_O]^+^, [oleanolic acid + H–COOH]^+^, and [oleanolic acid + H–COOH-H_2_O]^+^ ions, respectively. Therefore, compounds **63**–**66** were annotated as oleanolic acid-O-[O-methyl- glycuronoside], oleanolic acid -O-(O-acetyl pentose), anchusoside 1, and hederoside C, respectively^31^. Whereas compounds **89** and **91** were supposed to be oleanene triterpenoids. They exhibited their molecular ion peaks in positive ion mode at *m/z* 425.38 and 427.36, respectively. They both lost a methyl group to generate daughter peaks at *m/z* 410.35 and 412.33, respectively. Furtherly, compound **89** lost a carbonyl group to give a peak at *m/z* 382.34, while compound **91** lost a water molecule to produce a peak at *m/z* 394.31. Therefore, compounds **89** and **91** were annotated as oleanenone and oleanenol, respectively^32^.

##### Stigmastane derivatives

Two stigmastane derivatives (compounds **84** and **85**) with [M + H]^+^ ions at *m/z* 445.28 and 429.30, respectively, were identified in the mass spectra of *C. procera* extracts. They both lost a carbonyl group and [C_10_H_22_]^+^ ion to produce daughter peaks at *m/z* 417.27 and 303, for compound **84**; and at 401.29 and 287.02, for compound **85**. Compound **85** was found to be hydroxystigmastenone. Whereas compound **84** whose parent and daughter ions had extra 16 Da than those of hydroxystigmastenone was suggested to be hydroperoxystigmastenone^33^.

##### Ursane-type triterpenes

Ninteen ursane-type triterpenes (compounds **86–88, 90, 92–106**) were recognized in the mass spectra of *C. procera* extracts. Compound **87** with [M + H]^+^ ion at *m/z* 423.36 was annotated as calotroprocerol A. It exhibited a base peak at *m/z* 220 owing to retro Diels–Alder fragmentation. It also generated fragment ions at *m/z* 152, 220, 233 and 257 that proved the unsaturated nature of rings B and C. Whereas, the fragment ions at *m/z* 54, 67, 82, 121, and 136 suggested the saturated nature of rings D and E^34^.

Compounds **86** and **88** with parent peaks at *m/z* 421.44 and 425.38, were proposed to be calotroprocerone A and taraxadienol, respectively. They produced the same fragment ions generated from calotroprocerol A (compound **87**), except those generated from rings B & C that had 2 Da less in case of compound **86**, thus, proving the existence of one extra double bond due to the ketone functional group which presence was indicated by the fragment ion at *m/z* 28.01^29^. While in case of compound **88**, the fragment ions generated from rings B & C were 2 Da higher than those generated with calotroprocerol A indicating the lack of one double bond^29^. Moreover, compound **95** with [M − H]^−^ ion at *m/z* 465.23 was annotated as procerursenyl acetate. It was subjected to retro Diels–Alder fragmentation to generate daughter peaks at *m/z* 250 and 216. These fragment ions suggested the presence of an olefinic bond at C12 and acetoxy moiety in ring A/B. Additional fragment ions were generated at *m/z* 406 and 190 owing to [M-AcOH]^−^ and [250-AcOH]^−^ ions, respectively. Other fragment ions were also appeared due to loss of methyl groups^35^. Compounds **90, 92–94, 96–106** were annotated as Ursenone, Calotropeol, Methoxy ursene, Calotropursenyl acetate, α-Amyrin propanoate, α-Amyrin butyrate, α-Amyrin pentanoate, α-Amyrin caproate, α-Amyrin caprylate, α-Amyrin hydroxy cinnamate, α-Amyrin cinnamate, α-Amyrin undecanoate, α-Amyrin dodecanoate, α-Amyrin tetradecanoate, and α-Amyrin heptadecanoate, respectively, by comparison of their retention times and mass spectra with literature and reference standards. They all exhibited a fragment ion corresponding to the substituent attached to the ursenol skeleton. Then they give fragment ions that were related to methyl groups’ loss^36,37^.

#### Fatty acids

Compounds **107**–**110** displaying their parent peaks at *m/z* 255.15, 269.11, 283.22, and 449.39 were characterized as hexadecenoic acid and its methyl, ethyl, and tetradecyl esters, respectively. Hexadecenoic acid (compound **107**) demonstrated its fragment ions at *m/z* 237.13 and 211.14 due to neutral loss of H_2_O and CO_2_. Then it was subjected to sequential removal of CH_2_ groups^38^. Whereas compounds **108–110** generated their fragment ions at *m/z* 238 and 210 due to sequential removal of alkoxy and carbonyl groups. Afterwards, they lost CH_2_ groups, successively^38^. Compound **111** with [M + H]^+^ ion at *m/z* 439.27 is a fatty alcohol that was assigned as triacontanol. It lost a water molecule then underwent successive removal of CH_2_ groups^39^. While compound **112** with [M − H]^−^ ion at *m/z* 447.45 is an alkene that was annotated as dotriacontene. Its mass spectrum was characterized by peaks with 14 Da lower due to sequential degradation of CH_2_ groups^40^.

**Fig. 2:**
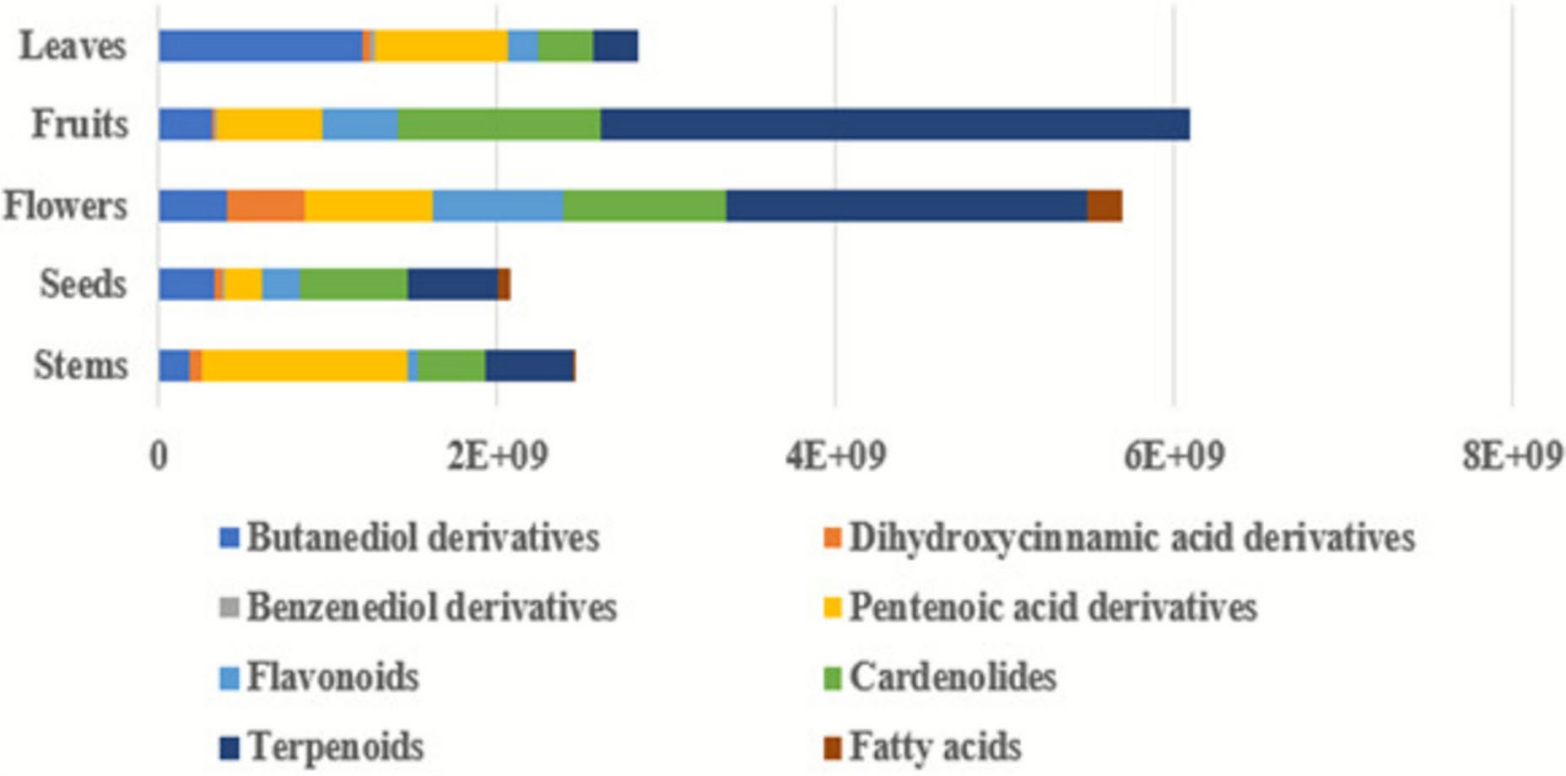
Mean relative percentile levels of the identified chemical classes in different *C. procera* organs.

The relative distribution of the aforementioned chemical classes in different *C. procera* organs was illustrated in Fig. 2. As shown in Fig. 2, terpenoids were the predominant chemical class in both the fruits and flowers of *C. procera*. Whereas, butanediol derivatives, cardenolides, and pentenoic acid derivatives were the major chemical classes in the leaves, seeds, and stems, respectively.

### Multivariate statistical analysis

#### Unsupervised pattern recognition of different *C. procera* organs by hierarchical clustering analysis (HCA)—heat map model

**Fig. 3:**
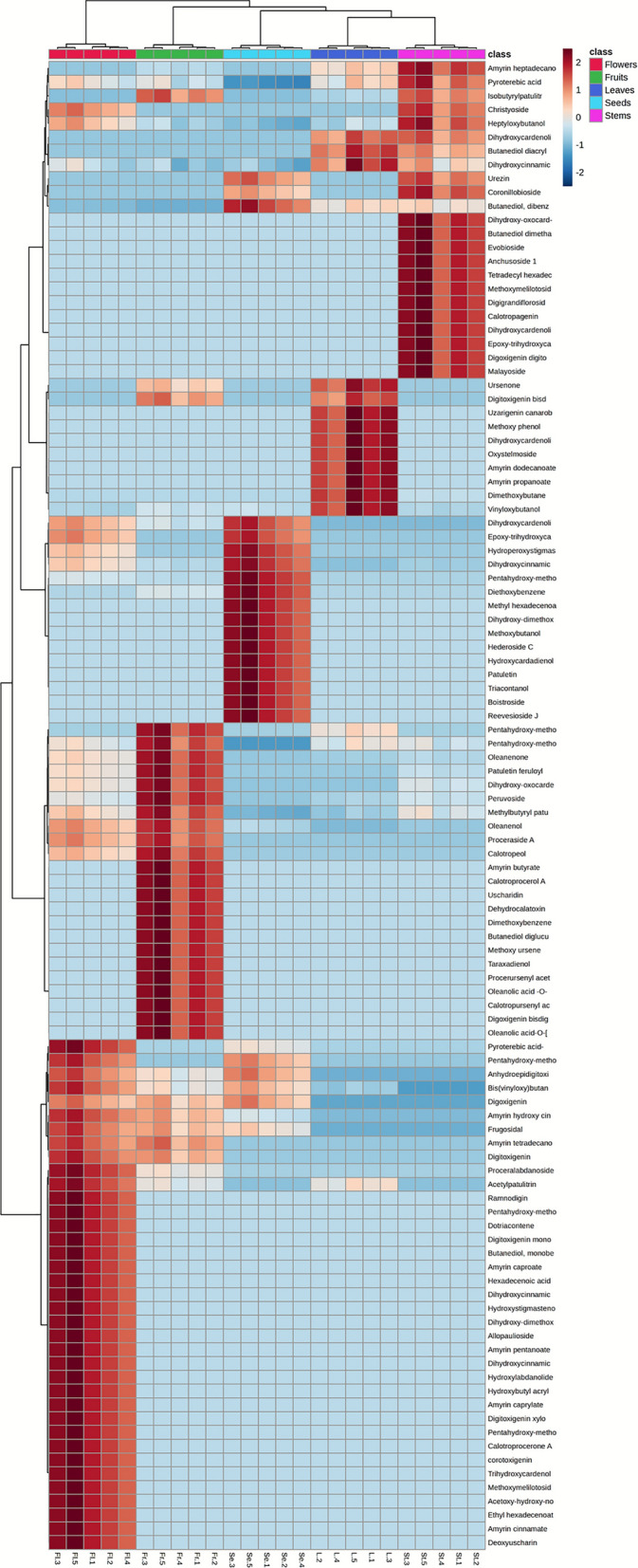
Heat map of identified compounds in different *C. procera* organ samples with the relative amounts of metabolites from low to high with a color gradient from dark blue (indicating low concentrations) to brick red (indicating high concentrations).

In this section, we compared the phytochemical profiles of five different parts of *C. procera* namely, flowers, fruits, leaves, seeds and stems. The analysis was performed using UPLC–MS/MS data combined with chemometric statistical techniques. As shown in Fig. 1, clear differences in the chemical composition were observed between the five organs. The dendritic hierarchical clustering analysis (HCA), together with the heat map (Fig. 3), separated the samples into five distinct clusters corresponding to flowers, fruits, leaves, seeds and stem samples. The heat map visually represented the distribution of identified compounds within these groups, with a color gradient from dark blue (indicating low concentrations) to brick red (indicating high concentrations). It was noted that the stem samples exhibited the highest levels of amyrinheptadecanoate, pyroterebic acid, isobutyrylpatulitrin, christyoside, heptyloxybutanol, evoboside, calotropagenin, malayosideurezin, coronillobioside and anchusoside 1 amongst others. Contrariwise, leaves samples showed higher concentrations of dihydroxycardenolide-O-( xylopyranosyl-allopyranoside), butanediol diacrylate, methyldihydroxycinnamate-di-methylether, uresenone, digitoxigenin bisdigitoxide, uzarigenin canarobioside, oxystelmoside, amyrindo decanoate, amyrin propanoate, dihydroxycardenolide-O-(xylopyranosyl-rhamnopyranoside) and others. In contrast, seeds samples had the highest levels of reevesioside J, boistroside, tricontanol, patuletin, hydroxycardadienol, acetyldihydroxycardenolide, epoxy-trihydroxycardenolide-methylether and hydroperoxystigmastenone. Additionally, fruits samples showed the greatest concentrations of proceraside, calotropeol, calotroprocerol A, uscharidin, dehydrocalatoxin, methoxyursene, taraxadienol, peruvoside, presurursenylacetate, calotropursenylacetate, amyrinbutyrate, digoxigenin digitoxoside and oleanenone. Finally, the flowers samples exhibited the highest levels of digoxigenin, frugosidal, proceralabdanoside, acetylpatulitrin, ramnodigin, dotriacontane, digitoxigenin monooctadioate, amyrin caproate, hexadecenoic acid, allopaulioside, amyrin pentanoate, hydroxylabdanolide, calotroprocerone A, corotoxigenin and deoxyuscharin as shown in Fig. 3.

#### Class discrimination of different *C. procera* organs using OPLS-DA model

**Fig. 4:**
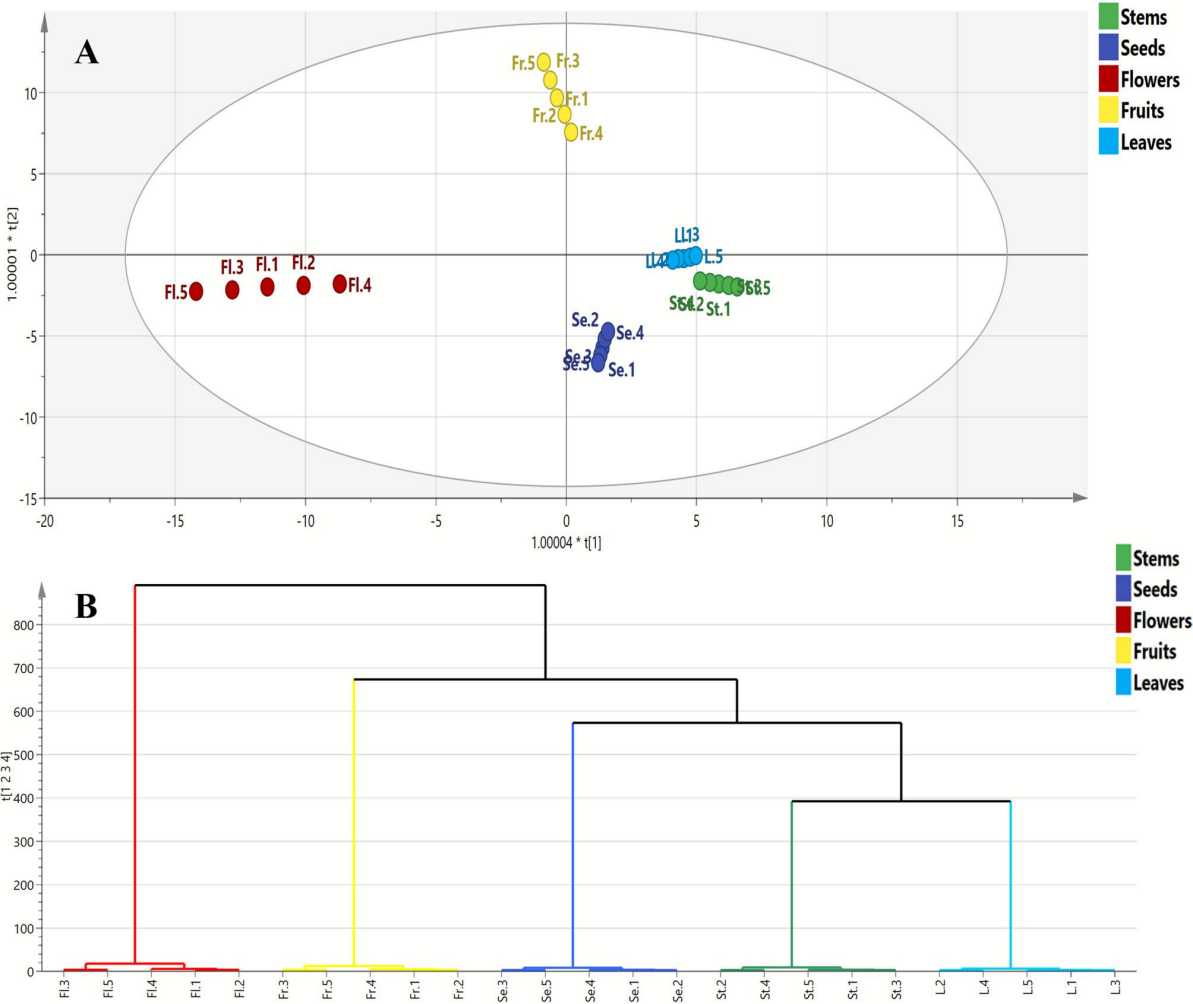
Orthogonal projections to latent structures discriminant analysis (OPLS-DA) score plot of the tested *C. procera* organ samples (**A**). Dendrogram derived from the hierarchical cluster analysis, based on the Ward method of flowers, fruits, leaves, seeds and stem samples (**B**).

The OPLS-DA (Orthogonal Projection to Latent Structures-Discriminant Analysis) model was used to refine dataset patterns and enhance the differentiation of Apple of Sodom samples. This model effectively highlighted both inter- and intra-class differences among *C. procera* flowers, fruits, leaves, seeds and stems by identifying unique chemical markers, as shown in coefficient plots for each organ (Fig. 4). The model’s reliability and predictive power were evaluated with R2Y (explained variance), which was 0.979, and Q2 (predicted variance), which was 0.971. Permutation plots were also generated to ensure the model was not overfitted (Fig. S1).

The OPLS-DA score scatter plot (Fig. 4A) demonstrated clear class separation along the first latent variable (LV1), with flowers and most fruit samples of *C. procera* clustering on the negative side, while leaves, stems and seeds samples were found on the positive side and closely clustered on lower right quartile, suggesting that the type of organ significantly influences the chemical profiles of *C. procera*. Likewise, the HCA results (Fig. 4B) were consistent with the score scatter plot results, showing a dendrogram with two main clusters: the first cluster for flower samples and the second cluster was further discriminated into two subclusters, one for fruit samples and the other subcluster was subsequently divided into seeds, leaves and stems. It was noted that leaves and stem samples were joined in one cluster indicating the proximity in their chemical composition.

**Fig. 5:**
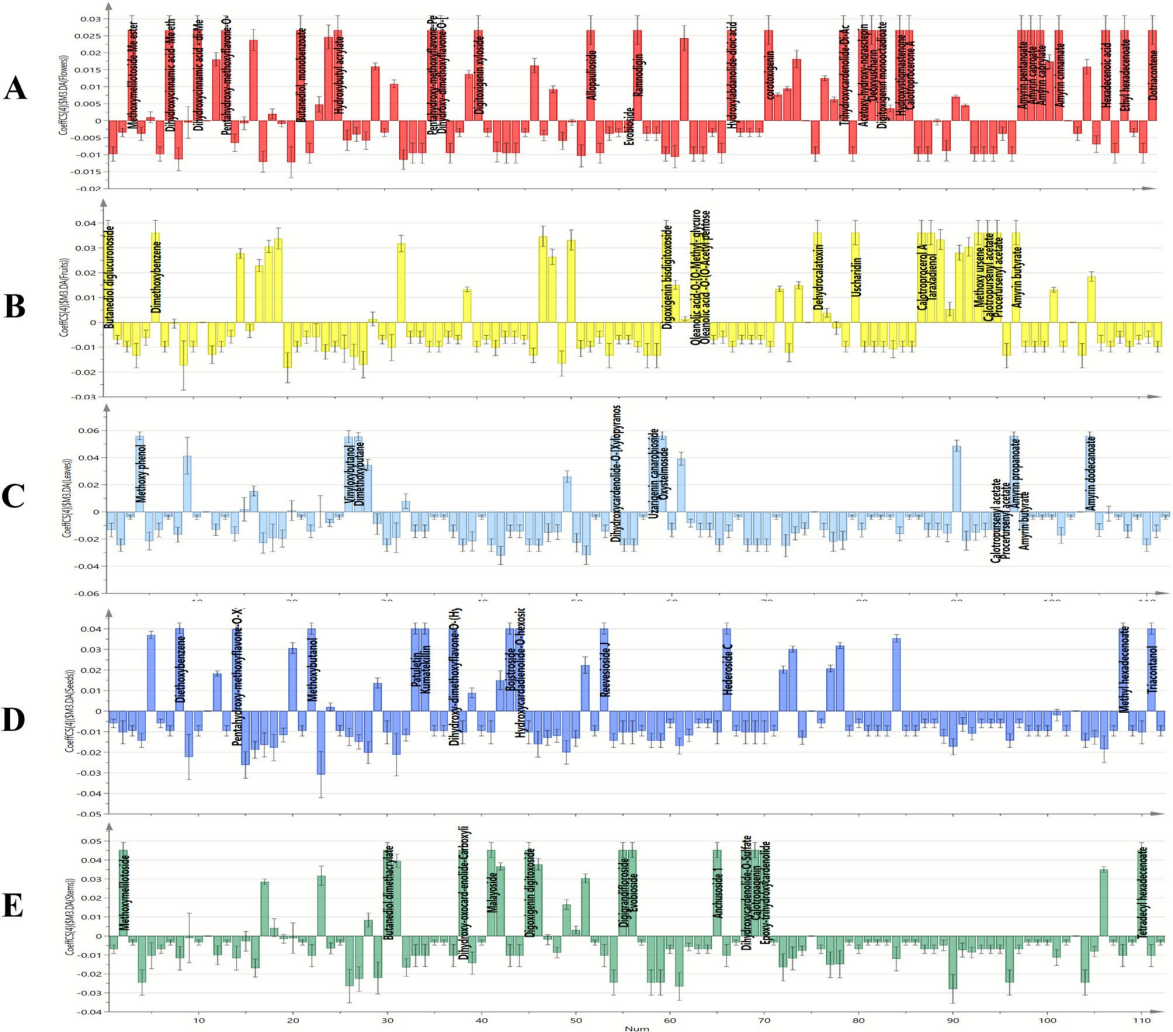
Coefficient plots of the OPLS-DA model for *C. procera* samples (**A**) flowers, (**B**) fruits, (**C**) leaves, (**D**) seeds and (**E**) stems samples.

Additionally, analysing the coefficient plots helped identify compounds that were positively correlated and contributed to distinguishing the different sample classes. The coefficient plot for flowers samples (Fig. 5A) highlighted that methyldihydroxycinnamate-dimethylether, butanediol dibenzoate, ramnodigin, corotoxigenin, digitoxigenin monooctadioate, amyrin pentanoate, hydroxylabdanolide-dioic acid, hydroxystigmastenone, amyrin caproate, hexadecenoic acid, methoxymelilotoside-methylester, dihydroxycinnamic acid-methylether, ethyldihydroxycinnamate acid-dimethylether, pentahydroxy-methoxyflavone-O- glucuronopyranoside, butanediol monobenzoate, hydroxybutyl acrylate, pentahydroxy-methoxyflavone-pentaacetate, dihydroxy-dimethoxyflavone-O-[hydroxymethyl-methyl-propenyl], digitoxigenin xyloside, allopaulioside, trihydroxycardenolide-diacetate, acetoxy-hydroxy-norasclepin, deoxyuscharin, calotroprocerone A, amyrin caprylate, amyrin cinnamate, ethyl hexadecenoate, dotriacontane were the key markers for this group. Meanwhile, the coefficient plot for fruit samples (Fig. 5B) revealed that uscharidin, taraxadienol, procerursenyl acetate, oleanolic acid-O-(O-methylglycuronoside), oleanolic acid-O-(O-acetyl pentose), methoxy ursine, dimethoxybenzene, digoxigenin bisdigitoxoside, dehydrocalatoxin, calotropursenyl acetate, calotroprocerol A, butanediol diglucuronoside and amyrin butyrate were the primary chemical markers for distinguishing this class. In contrast, the coefficient plot for leaves samples (Fig. 5C) showed that methoxy phenol, dihydroxycardenolide-O-(xylopyranosyl-rhamnopyranoside), uzarigenin canarobioside, oxystelmoside, amyrin propanoate and amyrin dodecanoate were the distinguishing markers for this class. Regarding the seeds samples class, its coefficient plot (Fig. 5D) revealed that diethoxybenzene, methoxybutanol, patuletin, kumatakillin, dihydroxy-dimethoxyflavone-O-(Hydroxy-methoxyphenyl) ether, boistroside, hydroxycardadienolide-O-hexoside, reevesioside J, hederoside C, methyl hexadecenoate and triacontanol were the discriminatory markers of this class. Finally, the coefficient plot of stems samples (Fig. 5E) demonstrated that methoxymelilotoside, butanediol dimethacrylate, dihydroxy-oxocardenolide-carboxylic acid-O-hexoside, malayoside, digoxigenin digitoxoside, digigrandifloroside, evobioside, anchusoside 1, dihydroxycardenolide-O-sulfate, calotropagenin, epoxy-trihydroxycardenolide and tetradecyl hexadecenoate were the key chemical markers for leaves samples class.

### Investigation of in vitro anti-inflammatory activities of *C. procera* organs’ extracts

**Fig. 6:**
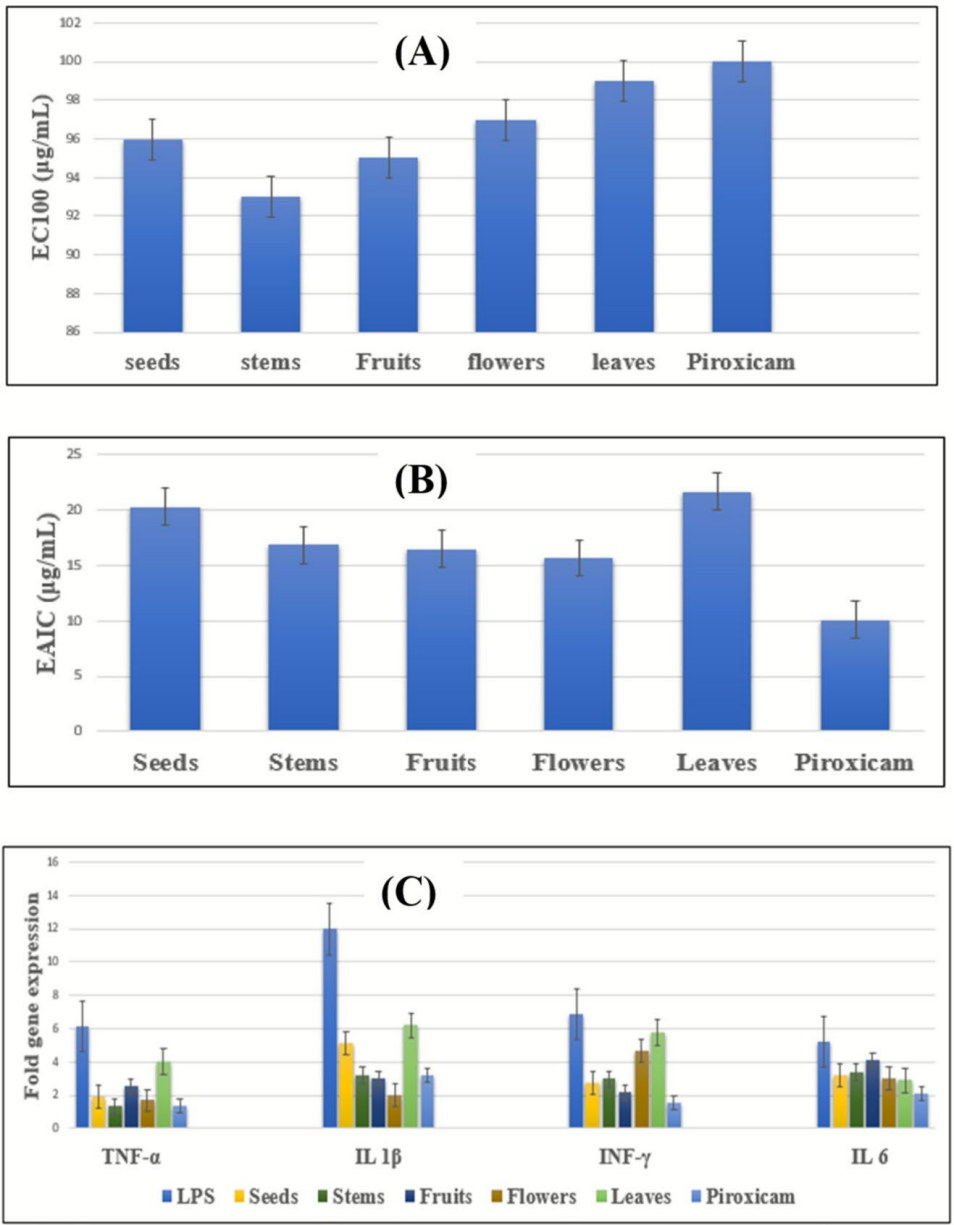
Bar charts showing the EC100 (µg/mL) (**A**), effective anti-inflammatory concentrations (EAICs) (µg/mL) of tested extracts (**B**) and the levels of TNF-α, IL-1β, IFN-γ, and IL-6 downregulation (expressed as fold change) of the studied samples (**C**).

At first, the safety of *C. procera*’s investigated extracts in addition to that of the standard anti-inflammatory drug (piroxicam) were assessed through calculating EC100 for them using MTT (3-(4,5-dimethylthiazol-2-yl) 2,5-diphenyltetrazolium bromide) assay. EC100 is defined as the extract’s concentration at which 100% of normal white blood cells (WBCs) remain viable^41^. It was observed that *C. procera*’s studied extracts, specially the leaves, flowers, and seeds extracts, had comparable EC100 values to that of piroxicam (100 µg/mL) denoting their high levels of safety (Fig. 6A).

After that, the anti-inflammatory efficacy of the examined extracts was compared to that of piroxicam through calculating their effective anti-inflammatory concentrations (EAICs). It was revealed that *C. procera* extracts possess comparable EAICs values to that exhibited by piroxicam (10.1 µg/mL) proving their promising inflammation suppression potentials. The extracts that showed the most auspicious results were those of the flowers, fruits, and stems with EAICs values of 15.67, 16.46, and 16.85 µg/mL, respectively (Fig. 6B).

Subsequently, polymerase chain reaction (PCR) was conducted to explore the impact of *C. procera* different organs’ extracts on the gene expression of some inflammatory mediators (TNF-*α*, IL 1*β*, INF-*γ*, and IL 6) in normal WBCs along with WBCs exposed to lipopolysaccharides (LPS). Lipopolysaccharides are vital constituents of the cell wall in gram-negative bacteria, which are fragmented into smaller units (lipid A, core protein, and O-antigen) that demonstrate significant immunogenic and pro-inflammatory characteristics^42^. TNF-*α*, IL 1*β*, INF-*γ*, and IL 6 are inflammatory mediators that play key roles in the immune response and are involved in the regulation of inflammation. These cytokines can promote inflammation and are often elevated in various inflammatory conditions^43^.

Our study demonstrated that LPS elevated TNF-*α*, IL-1*β*, INF-*γ*, and IL-6 genes’ expression levels by 6.12, 11.97, 6.84, and 5.2 times, respectively. It was noted that *C. procera* extracts brought the expression of these upregulated genes down to levels similar to what was observed with piroxicam (Fig. 6C). Intriguingly, it was remarked that the fruits and flowers extracts of *C. procera* exhibited more downregulation to IL-1*β* gene’s elevated level to 3.05 and 1.99 folds, respectively, which are lower than that demonstrated by piroxicam (3.2 folds), indicating their proficient inflammation inhibitory efficacy (Fig. 6C).

### Identification of bioactive metabolites associated with anti-inflammatory activity using OPLS model

To explore how the type of Apple of Sodom organ influences anti-inflammatory activity and identify relevant biomarkers, x variables (UPLC/MS–MS data) and y variables represented by inflammatory markers (TNF-*α*, IL-1*β*, INF-*γ* and IL-6) were incorporated into the OPLS model. The model showed a high R2Y value of 0.952, indicating strong explained variance, and a Q2 value of 0.919, suggesting excellent predictive performance, thus confirming the model’s reliability. Permutation plots were also created to ensure that the model was not overfitted (Fig. S2).

**Fig. 7:**
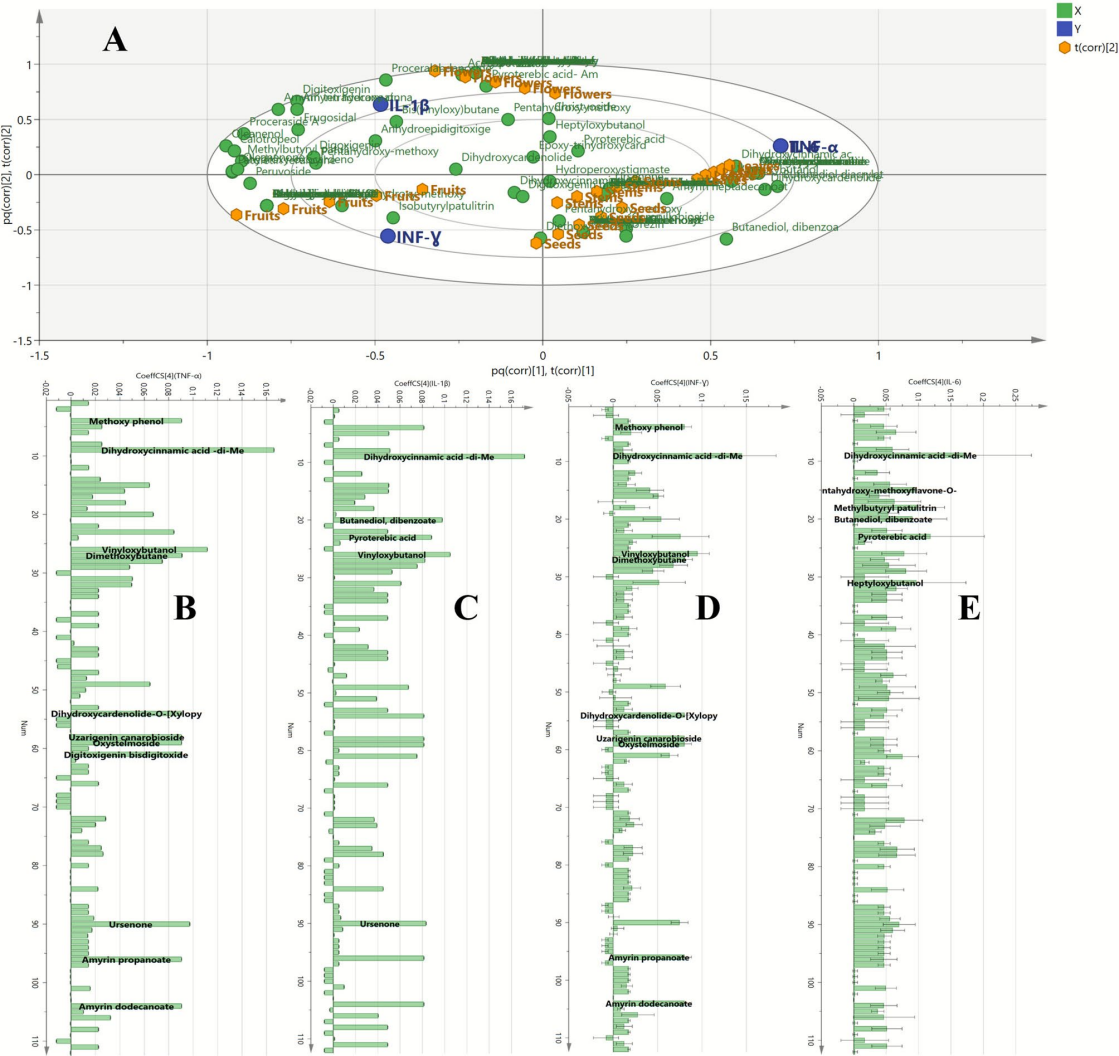
Orthogonal projections to latent structures (OPLS) biplot of the tested *C. procera* samples in correlation to the tested inflammatory mediators (**A**) and Coefficient plot showing correlation analysis between potentially active metabolites and TNF-*α* (**B**), IL 1*β* (**C**), INF-*γ* (**D**), and IL 6 (**E**).

The biplot (Fig. 7A) displayed the spatial relationships between the *C. procera* organs’ samples and their respective biological activities, highlighting the bioactive markers responsible for anti-inflammatory effects. A strong spatial correlation was found between flowers samples and IL-1*β* proinflammatory markers, as both appeared in the same quartile of the OPLS biplot. In the same manner, leaves samples showed the greatest correlation with IL-6, while stems samples were closely spaced to TNF-*α* marker. Finally, the fruits samples were the closely allocated to INF-*γ.*

The coefficient plot (Fig. 7B) revealed bioactive metabolites that positively impacted TNF-α gene suppression. These included methyldimethoxycinnamate, vinyloxybutanol, ursenone, dimethoxybutane, uzarigenin canarobioside, oxystelmoside, methoxy phenol, dihydroxycardenolide-O-(xylopyranosyl-rhamnopyranoside), amyrin propanoate, amyrin dodecanoate and digitoxigenin bisdigitoxide, which were identified as key contributors to this activity. On the other hand, methyldimethoxycinnamate, vinyloxybutanol, butanediol dibenzoate, pyroterebic acid and ursenone were highly correlated with IL-1β suppression as shown in (Fig. 7C). Meanwhile, the pro-inflammatory marker INF-*γ* was positively affected by methyldimethoxycinnamate, vinyloxybutanol, dimethoxybutane, methoxy phenol, dihydroxycardenolide-O-(xylopyranosyl-rhamnopyranoside), uzarigenin canarobioside, oxystelmoside, amyrin propanoate and amyrin dodecanoate as depicted in (Fig. 7D). Finally, methyldimethoxycinnamate, pyroterebic acid, methylbutyryl patulitrin, heptyloxybutanol, pentahydroxy-methoxyflavone-O-deoxyhexoside and butanediol dibenzoate were the positive contributors to IL-6 suppression (Fig. 7E).

Some of these revealed bioactive markers align with previous studies that demonstrated that dimethoxycinnamic acid moiety exhibited a heightened anti-inflammatory effect in reducing carrageenan-induced rat paw edema, showing activity similar to that of NSAIDs (non-steroidal anti-inflammatory drugs)^44^. It also caused down-regulation of iNOS and TNF-α biomarkers^45^. Additionally^46^, demonstrated that amyrin derivatives exerted significant anti-inflammatory effects by inhibiting LPS-induced IL-1β, IL-6, TNF-α secretion in THP-1 cells (which are a human monocytic cell line derived from an acute monocytic leukemia patient) and hold promise as an alternative topical treatment for osteoarthritis and inflammatory disorders, potentially with fewer side effects than NSAIDs. Regarding cardenolides and cardiac glycosides, they exhibited protective effect on inflammation produced by doxorubicin-induced cardiotoxicity in mice exemplified by lowering IL-6, TNF-α and CRP (C-reactive protein)^47^. Moreover, cardiac glycosides primarily inhibit cell proliferation and the secretion of proinflammatory cytokines from leukocytes. They have been also found to decrease inflammatory symptoms in different animal models of acute and chronic inflammation^48^. In addition, uzarigenin showed resemblance in several in silico drug-likeness parameters with the synthetic drug ibuprofen which is a well-established analgesic and anti-inflammatory agent^49^. On the other hand, Santos et al. have reported that terebic acid and its derivatives possessed anti-inflammatory activity^50^.

## Conclusion

This study demonstrates a comprehensive framework that combines UPLC–MS/MS-based metabolomics, multivariate statistical analysis, and in vitro inflammation inhibition analysis to explore the chemical variability among different *C. procera* organs and to assess their anti-inflammatory potential for the first time. The study revealed that *C. procera* possesses significant phytochemical diversity across its various organs’ extracts, with distinct profiles that contribute to its traditional medicinal uses. The identification of 112 phytoconstituents, including butanediol, benzenediol, dihydroxycinnamic acid, pentenoic acid derivatives, flavonoids, terpenoids, cardenolides, and fatty acids underscores the plant’s rich chemical complexity and potential therapeutic applications.

The anti-inflammatory assays revealed that *C. procera* extracts had comparable EC100 and EAICs values to that of piroxicam denoting their high levels of safety and inflammation suppression efficacy. They also demonstrated that extracts from the flowers and fruits exhibited notable efficacy in downregulating pro-inflammatory cytokines, particularly IL-1β, suggesting their promise as natural anti-inflammatory agents. These findings align with traditional uses of *C. procera* in managing inflammatory conditions, providing a scientific basis for its application in herbal medicine.

Further research is warranted to elucidate the specific mechanisms of action of these recognized bioactive compounds and evaluate the safety and efficacy of *C. procera* in clinical settings. This will pave the way for the development of novel therapeutic strategies utilizing *C. procera*'s medicinal properties, potentially offering safer alternatives to synthetic anti-inflammatory medications.

## Supplementary Information

Supplementary Information.

## Data Availability

Data is provided within the manuscript or supplementary information files.
