# Supplementary material for: Unveiling the metabolic profile and anti-inflammatory potential of apple of Sodom (Calotropis procera (Aiton) W.T.) by UPLC–MS/MS chemometric analysis
Source: Sci Rep. 2026 Feb 6;16:5402. doi: 10.1038/s41598-025-33542-1 (PMC12886814; doi:10.1038/s41598-025-33542-1)
Supplement: Supplementary file 1 — Supplementary Information. [file 41598_2025_33542_MOESM1_ESM.docx]

**Unveiling the Metabolic Profile and Anti-inflammatory Potential of Apple of Sodom (*Calotropis procera* (Aiton) W.T.): A Comprehensive UPLC-MS/MS Chemometric Analysis of Organ-Specific Extracts**

**Supplementary file**

**2. Experimental:**

**2.4. Anti-Inflammatory Activity Assessment of Different *C. procera* Organs Extracts**

**2.4.1. Isolation and cultivation of human white blood cells**

**Procedure**

In a sterile heparin tube, a blood specimen was obtained then 1ml blood was taken into 15ml centrifuge tube then the tube was filled to capacity with fresh cold lysing solution. After that, the tube is inverted for about 10 minutes at room temperature until the liquid became clear red. Centrifugation of the blood specimen was carried out at 4^o^ C for 10 min at 2000 rpm followed by decantation of the supernatant and draining of the tubes was done. Suspending of the pellets (WBCs) was in 10 ml cold PBS followed by centrifugation. Then pellets were resuspended in RPMI culture medium. Dye exclusion method was used for the assessment of WBCs viability and counting (Louis & Siegel, 2011). Mixing fifty microliters of cell suspension with an equal volume of 0.5% trypan blue staining solution was done followed by its loading onto hemocytometer. Finally, counting of viable "unstained" and nonviable "stained" cells was carried out in each of the four corner quadrants (A, B, C, D).

**Calculation**

**N / ml = mean of WBCs counting x10^4^ × D**

N: Number of viable or nonviable cells

D: Sample dilution (1:1 with the trypan blue).

**% Cell viability = Number of viable cells × 100**

**Total number of cells**

In order to use the cells for assays, at least 90% of the cells must be viable. Culturing of WBCs was in RPMI media, then they incubated in CO_2_ incubator for six days. Seeding of WBC's was 100,000 cells/ well (96 well cell culture plate). The incubation conditions in CO_2_ incubator were 37°C temperature, 5% CO_2_, and 90% relative humidity.

**2.4.2. Assessment of cytotoxicity of *C. procera* different organs extracts compared to** **piroxicam**

**Procedure**

In this assay, 200 µl of cultured medium containing 100,000 WBCs / well were treated with different concentrations (0, 31.25, 62.5, 125, 250 and 500 μg/ml) of the crude extracts in RPMI medium without fetal bovine serum or piroxicam (standard anti-inflammatory drug. After that, plates were incubated for 72 h in CO_2_ incubator and conditions were 37°C temperature, 5% CO_2_, and 90% relative humidity). After incubation, 20μl of MTT solution was added to each well followed by plates’ incubation in order to allow MTT reaction to be performed. Then, centrifugation of plates was carried out at 1650 rpm for 10 min followed by discarding the medium. MTT byproduct (the formazan crystals) were re-suspended in 100 μl DMSO. The absorbance was measured at a wavelength of 570 nm using optima spectrophotometer for detecting safe dose, which cause 100% cell viability.

**The % viability was calculated as follow: (A_T_-A_b_ /A_C_-A_b_) x 100**

**A_T_** = mean absorbances of cells treated with different concentration of each plant extract

**A_C_** = mean absorbances of control untreated cells with culture medium only

**A_b_**_=_ mean absorbances of cells treated with vehicle of plant extract (RPMI without fetal bovine serum)

The cytotoxicity assay of the compound was expressed as EC_100_, and was calculated by the Graphpad Instat software using the % viability calculated from the serial dilutions of each plant extract.

**2.4.3. Detection of the effective anti-inflammatory concentrations (EAICs) of the used treatments in lipopolysaccharides (LPS)-stimulated human WBC's culture**

**Procedure**

In a 96 well plat, A volume of 50 μl of the culture medium that contained 100,000 of human WBCs was dispensed per well. The inflammation was induced by adding 50 μl of LPS to the plated cells and incubated in CO_2_ incubator. After 24 h, the plate was centrifuged at 1650 rpm for 5 min and the supernatants were discarded and then 200 µl of serial concentrations (0, 31.25, 62.5, 125, 250 and 500 μg/ml in culture media) of the crude extracts or the standard anti-inflammatory drugs piroxicam were added. The control cells contained cell culture medium only. The plates were incubated for additional 72 h in CO_2_ incubator. After 72 h of incubation, the cell proliferations were measured using MTT (as previously illustrated). Stimulation index (SI) was used to assess the cell proliferations.

**Stimulation index** = (mean absorbance of LPS-stimulated cells or LPS-stimulated cells treated with different concentrations of natural product / absorbance of control untreated cells).

The effective anti-inflammatory concentration (EAICs) of each extract that were able to bring back the abnormal proliferation of LPS-stimulated cells to normal proliferation of control untreated cells (SI = 1) were calculated using Instate graph pad.

**2.4.4. Extraction of RNA of untreated and treated LPS-stimulated human white blood cells**

**Procedure**

In 50 µl of solution R1, cell pellets were then suspended and mixed for 30 s, then their incubation was done at room temperature for 1 min. Three hundred microliters of solution R2 were added and mixed for 30 s then the centrifugation was done at 4ºC for 3-5 min. Into a spin column the supernatant was transferred and centrifuged for 30 s at 14000 rpm at 4ºC. Three hundred microliters of working wash buffer were added into the spin column after discarding the flow-through and centrifuged for 30 s. This step was repeated twice. Centrifugation of the spin column was done for 1min at 10,000 rpm then it was transferred to a sterile 1.5 ml micro centrifuge tube. To the central of the membrane, 30 µl of elution buffer were added and incubated at room temperature for 1 min followed by their centrifugation for 30 s at 14000 rpm at 4ºC. Finally, determination of the optical density (OD) of the extracted RNA was done through measuring the absorbance and purity at A260 and A260/A280 nm, respectively using spectrophotometer and kept in -80°C until real time PCR.

**2.4.5. cDNA synthesis from RNA extracted from untreated and treated LPS-stimulated human white blood cells**

**Procedure**

Two µg of total RNA or nuclease-free water and 1 µl of oligo dT primer were added to nuclease-free water in a total volume of 12 µl in PCR tubes, then they mixed gently. Centrifugation, incubation at 65ºC for 5 min in PCR machine was carried out then they placed back on ice immediately. The gentle mixing of 4 µl of 5X reaction buffer, 1 µl of RNase inhibitor, 2 µl of dNTPs mix and 1 µl of reverse transcriptase or 1 µl of nuclease-free water instead of reverse transcriptase for reverse transcriptase negative control with previous mixture was performed. After that, spin down and incubation for 60 min at 42ºC followed by heat inactivation at 70ºC for 5 min in PCR machine was carried out.

**2.4.6. Determination of IL-1*β*, IL 6, TNF and INF-*γ* expression level by real time polymerase chain reaction (PCR)**

**Procedure**

Thirteen µl of 2 X SYBR green master mix was mixed with 5µl of cDNA, 0.5 µl of 10 pmoles/ml forward primer and 0.5 µl of pmoles/ml reverse primer for each primer in PCR tubes. As for the reference tube, 0.5 µl of 10 pmoles/ml forward primer of β-actin and 0.5 µl of 10 pmoles/ml for reverse primer of β- actin were added. In order to assess for reagent contamination or primer dimers, another tube was used as a non-template control (NTC) by adding 1 µl of nuclease-free water instead of template used. After that, the gentle mixing of the tubes with 6.5 µl nuclease free water without creating bubbles was done and then spinned for few seconds. In the cycler, samples were placed and the program was started as following; initial denaturation (1 cycle of 95ºC for 10 min), followed by denaturation (40 cycles of 95ºC for 15 sec), annealing (at 60ºC for 30s) and extension (at 72ºC for 30s). the effect of LPS and extracts on gene expression was expressed as Fold change in gene expression which calculated according the following equations:

**Calculation**

**Expressions fold levels of gene calculated by**

ΔCt _normal_ =Ct _normal untreated cells_ – Ct _reference_

ΔCt _tested plant extract_ = Ct _tested plant extract-treated cells_ – Ct _reference_

ΔCt _induced_ =- Ct _LPS-exposed cells_ – Ct _reference_

**In case of genes:**

ΔΔCT _tested plant extract_ = ΔCt _tested plant extract_– ΔCt _normal_

ΔΔCT _induced_ = ΔCt _induced_ – ΔCt _normal_

**In case of GAPDH:**

ΔΔCT _tested plant extract_ = ΔCt _normal_ - ΔCt _tested plant extract_

ΔΔCT _induced_ = ΔCt _normal_ - ΔCt _induced_

**Fold change in gene expression = log (2^-ΔΔCT^)**

**Where:**

**Ct _tested plant extract_**: threshold cycle value of genes of extracted mRNA of plant extract treated-LPS-stimulated WBCs which is defined as the cycle number at which the fluorescence generated within a reaction crosses the fluorescence threshold.

**Ct reference**: threshold cycle value of GAPDH which is used for normalization.

**Ct _normal_**: threshold cycle value of genes of extracted mRNA of untreated control WBCs

**Ct _induced_**: threshold cycle value of gene of extracted mRNA of LPS-stimulated WBCs

The primers used:

| TNF-*α* | F-CTCTTCTGCCTGCTGCACTTTG |
| --- | --- |
|  | R- ATGGGCTACAGGCTTGTCACTC |
| IL-6 | F, 5′-TGAACTCCTTCTCCACAAGCG-3′ |
|  | R, 5′-TCTGAAGAGGTGAGTGGCTGTC-3′ |
| IL-1*β*, | F, CCACAGACCTTCCAGGAGAATG |
|  | R, GTGCAGTTCAGTGATCGTACAGG |
| INF-γ | F, GAGTGTGGAGACCATCAAGGAAG |
|  | R, TGCTTTGCGTTGGACATTCAAGTC |
|  | R, GGAAGATGGTGATGGGATT |
| GAPDH | F, GGATTTGGTCGTATTGGG |
|  | R, GGAAGATGGTGATGGGATT |

**References:**

Louis, K. S., & Siegel, A. C. (2011). Cell viability analysis using trypan blue: manual and automated methods. *Methods in Molecular Biology (Clifton, N.J.)*, *740*, 7–12. https://doi.org/10.1007/978-1-61779-108-6_2

**3. Results and discussion:**

**3.1. The use of UPLC-MS/MS for characterization of phytoconstituents in different *C. procera* organs’ extracts:**

**3.1.5. Flavonoids:**

**3.1.5.1. Flavonol secondary glycosides:**

For the secondary glycosides, Peak **11** was identified as dihydroxy-dimethoxyflavone-O-[ deoxyhexosyl-hexoside], with a protonated ion peak at 623.5 Da. Daughter ions were observed at 461 Da, attributed to the loss of a hexose moiety, and at 315 Da, indicative of the loss of a deoxy sugar moiety. Moreover, daughter ions appeared at 287 Da, corresponding to the loss of a CO group, and at 300 Da and 285 Da, attributed to the loss of two methyl groups. Regarding flavanol 3-O glycosides, peak **12** was identified as pentahydroxy-methoxyflavone-O-sulfate, O-hexoside, with a protonated ion peak at 575.6 Da. Fragment ions at 413 Da and 333 Da are attributed to the loss of the SO_3_ group and a hexose sugar respectively. Peaks **13** and **15** were identified as pentahydroxy-methoxyflavone-O-glucuronopyranoside and pentahydroxy-methoxyflavone-O-deoxyhexoside, with quasi-molecular ion peaks [M+H]^+^ at 509.4 Da and 479.2 Da, respectively. Both compounds exhibited identical MS^2^ fragments of 333 Da attributed to the loss of a hexose moiety for the first compound and a deoxy sugar for the second one. Similarly, peak **14** was tentatively annotated as pentahydroxy-methoxyflavone-O-xyloside, with a deprotonated ion peak at 463.4 Da and MS^2^ ion at 331 Da resulting from the loss of a pentose sugar. Peak **19** was identified as patuletin feruloylhexoside with a molecular ion peak [M-H]^-^ at 669.4 Da and daughter ion peak at 331 Da due to the loss of hydroxyl-methoxy-cinnamoyl hexose (Qiao et al., 2012). Additionally, compounds **12**, **13**, **14**, **15**, and **19** showed ion peaks at 318 Da and 305 Da in positive ion mode and 316 Da and 303 Da in negative ion mode due to the loss of a methyl group and a CO group.

**3.1.5.2. Flavonol primary glycosides:**

Regarding, 7-O flavonol glycosides, peak **16** was assigned as acetylpatulitrin, featuring a protonated ion peak at 537.4 Da. Its mass spectrum presented a fragment ion at 333 Da due to the loss of the acetyl glucose moiety (Parejo et al., 2004). Peak **17**, tentatively identified as isobutyrylpatulitrin, displayed a deprotonated ion peak at 563.4 Da. Fragment ion was observed at 331 Da resulting from the loss of methyl propanoyl hexose (Heilmann et al., 1999). Peak **18**, recognized as methylbutyryl patulitrin, showed a quasi-molecular ion peak [M-H] ^-^ at 577.4 Da. Its daughter ions included 331 Da due to the loss of methyl butanoyl hexose. Similarly, the three compounds showed peaks at 318 and 305 Da in positive ion mode and at 316 and 303 Da in negative ion mode, indicative to the loss of a methyl group and a CO group, respectively.

**3.1.5.3. Flavonol aglycones:**

For flavonol aglycones, peak **32** was identified as pentahydroxy-methoxyflavone-O-sulfate, presenting a protonated ion peak at 413 Da. A fragment ion appeared at 333 Da attributed to the loss of the SO_3_ group. Peak **33** was identified as patuletin with a deprotonated ion peak at 331.3 Da. Additionally, peak **35** was identified as pentahydroxy-methoxyflavone-penta-Ac with a deprotonated ion peak at 541.5 Da. Fragment ions were observed at 499 Da, 457 Da, 415 Da, 373 Da, and 331 Da, attributed to the loss of five acetyl groups. Furthermore, daughter ions were observed in the mass spectrum of compounds **32**, **33** and **35** at 318 Da and 305 Da in positive ion mode and at 316 Da and 303 Da in negative ion mode, indicative to the loss of a methyl group and a CO group respectively (Fabre et al., 2001). Peak **34** was identified as kumatekillin, featuring a parent ion peak [M-H]^-^ at 313.3 Da. Its mass spectrum showed a fragment ion at 285 Da, attributed to the loss of a CO group, and at 298 Da and 283 Da, indicative of the loss of two methyl groups (Zhang et al., 2017). Peak **36** was tentatively identified as dihydroxy-dimethoxyflavone-O-[hydroxymethyl-methyl-propenyl], with a pseudo-molecular ion peak [M+H]^+^

at 399.3 Da. MS^2^ fragments were observed at 315 Da, 300 Da, 285 Da and 287 Da, attributed to the loss of the [hydroxymethyl-methyl-propenyl] group, followed by two methoxy groups, and finally a CO group. Peak **37** was identified as dihydroxy-dimethoxyflavone-O-(hydroxy-methoxyphenyl) ether, with a deprotonated ion peak at 435.4 Da. Daughter ions appeared at 313 Da, 298 Da and 283 Da indicative of the loss of the hydroxy-methoxyphenyl group, followed by two methyl groups, and then a CO group.

**3.1.6. Cardenolides:**

**3.1.6.1. Cardenolides genins:**

Regarding the cardenolides genins, two peaks represent oxacardenolides (peaks **69** and **71**) featuring an aldehyde group at carbon number 19, while the remaining seven peaks denote cardenolide skeletons with a methyl group attached to carbon number 19 (peaks **68**, **72, 74, 75**, **77, 78** and **79**) (Josephs et al., 2010). Peak **69** was identified as calotropagenin. Such identification was based on [M-H]^-^ ion peak at *m/z* 403.4 and daughter peaks at [M-H-H_2_O]^-^ 385 Da, [M-H-2H_2_O]^-^ 367 Da, 349 [M-H-3H_2_O]^-^, and [M-H-2H_2_O-CO]^-^ 339 Da, due to stepwise elimination of hydroxyl groups and the carbonyl group. The fragmentation pattern closely resembles that of strophanthidin (Filigenzi et al., 2004), differing only in the position of one hydroxyl group (Kanojiya & Madhusudanan, 2012). Furthermore, peak **71** was tentatively annotated as corotoxigenin (ABE et al., 1992), which is one hydroxyl group less than compound **69**. This determination was established by a mass peak at [M+H]^+^ 389.3 Da and fragment ion peaks at [M+H-H_2_O]^+^ 371 Da, [M+H-2H_2_O]^+^ 353 Da, and [M+H-H_2_O-CO]^+^ 343 Da.

For cardenolides with a methyl group at carbon 19, peak **72** was determined to be digoxigenin, discerned by the pseudo-molecular ion at *m/z* 391.3 and subsequent MS^2^ fragments at *m/z* 373, 355, and 337 resulting from sequential dehydration of three hydroxyl groups (Ravi et al., 2020). By referring to literature, peaks **74** and **75** were identified as digitoxigenin and its isomer. They both share the same quasi-molecular ion [M+H] ^+^ at *m/z* 375.3. Notably, both compounds possess one fewer hydroxyl group compared to digoxigenin (peak **72**). They both show daughter ions at 357 Da and 339 Da due to the stepwise elimination of two water molecules (Ravi et al., 2020; Y. Singh et al., 2021). Peak **78**, identified as dihydroxycardenolide-Ac, is the acetylated version of digitoxigenin (peak **74**). It revealed a pseudo-molecular ion peak at *m/z* 415.4, accompanied by daughter peaks at *m/z* 373, 355 and 337, attributed to the loss of the acetyl group and sequential loss of water molecules, respectively. While peak **79**, tentatively identified as trihydroxycardenolide-Di-Ac, displayed a parent ion at *m/z* 473.4, with fragment ions at *m/z* 431, 389, 371, 353, and 335, attributed to the loss of two acetyl groups followed by three water molecules. This compound corresponds to the diacetylated form of digoxigenin (peak **74**). However, peak **68** was identified as the sulphated derivative of digitoxigenin. It was determined to be dihydroxycardenolide-O-sulfate, discerned by the pseudo-molecular ion [M-H] ^-^ at *m/z* 453.4 and subsequent daughter peaks at *m/z* 373, 355 and 337 resulting from loss of a sulphate group and sequential dehydration of two hydroxyl groups.

Compound **83** was identified as digitoxigenin monooctadioate showing a deprotonated ion peak at *m/z* 529.5 and daughter peaks at *m/z* 373, 355 and 337 due to the loss of octadionate group and two water molecules. Moreover, compound **77** was identified as anhydroepidigitoxigenin. It showed protonated ion peak at *m/z* 357.3 and daughter ion at *m/z* 339 due to the loss of water molecule from the molecular ion (Huq et al., 1999; Shaker et al., 2010). This compound represents the C14-dehydrated form of digitoxigenin, which could be formed biogenetically by dehydration (Y. Singh et al., 2021). Additionally, compound **70** was identified as epoxy-trihydroxycardenolide showing a protonated ion peak at *m/z* 405.2 and daughter ion peaks at *m/z* 373, 355 and 337 due to the loss of three water molecules. Compound **73** was identified as epoxy-trihydroxycardenolide-Me ether showing a deprotonated ion peak at *m/z* 417.4 and daughter peaks at *m/z* 373, 355 and 337 due to the loss of three water molecules (Y. Singh et al., 2021).

**3.1.6.2. Cardenolides Primary glycosides:**

Out of the 24 cardenolide glycosides, 13 compounds (peaks **38**-**41**, **43**-**48**, **52**, **53** and **57**) were identified as primary glycosides, with eight of them featuring oxacardenolide aglycones (peaks **38**, **39**, **41**, **43**, **46**-**48** and **52**). Peak **38**, identified as dihydroxy-oxocard-enolide-carboxylic acid, O-hexoside, displayed a molecular ion peak at 565.4 Da. Daughter ions were observed at [M-H-162]^-^ 403 Da, 385 Da and 367 Da, which were attributed to the loss of a hexose sugar and the successive loss of two water molecules. Meanwhile, peak **39** was identified as frugosidal displaying a protonated ion peak at 551.7 Da. A fragment peak was observed at [M+H-146]^+^ 405 Da, attributed to the loss of a deoxy hexose moiety, moreover, ion peaks at 387 Da, 369 Da and 351 Da were observed arising from the loss of three water molecules (Pederson et al., 2020). Peak **41**, sharing the same aglycone as compound **39**, was identified as malayoside, presenting a pseudo-molecular ion peak at 535.4 Da. Additionally, fragment ion peaks were detected at [M+H-146]^+^ 389 Da, 371 Da and 353 Da, indicating the loss of a deoxy hexose sugar moiety and two water molecules (Hu et al., 2021). Boistroside (peak **43**), a compound similar to malayoside (peak **41**) but with a different sugar moiety, exhibited a protonated ion peak at 519.4 Da and daughter ion peaks at [M+H-130] ^+^ 389 Da, attributed to the loss of dideoxy hexose, as well as peaks at 371 Da and 353 Da, corresponding to the loss of two water molecules (Marshall, 1964). Peaks **46** and **47** exhibited identical pseudo-molecular ion peaks at 549.4 Da. They also share the same aglycone structure as malayoside, with daughter ion peaks observed at [M+H-160]^+^ 389 Da, 371 Da, and 353 Da. These daughter ion peaks were attributed to the loss of a deoxy methyl hexose moiety and two water molecules. Peak **46** was identified as christyoside, while peak **47** was identified as peruvoside (Abe et al., 1992). Additionally, peak **48**, identified as proceraside A, displayed a molecular ion peak at 575.4 Da. Fragment ions were observed at [M-H-188]^-^ 387 Da, 369 Da and 351 Da, which were attributed to the loss of acetyl deoxy hexose sugar and successive loss of two water molecules (Ibrahim et al., 2014). Peak **52** was identified as allopaulioside, featuring a protonated ion peak at 533.6 Da. It displayed molecular ion peaks at [M+H-144]^+^ 389 Da, associated with the loss of dideoxy methy hexose, and MS^2^ fragment ions at 371 Da and 353 Da, arising from the loss of two water molecules (Hill et al., 1991).

For glycosides containing an aglycone with a methyl group at carbon 19, peak **40** was identified as digitoxigenin xyloside, revealing a deprotonated ion peak at 505.4 Da. Daughter ion peaks were observed at [M-H-132]^-^ 373 Da, 355 Da, and 337 Da, attributed to the loss of a pentose sugar and two water molecules (Butler et al., 2014). Whereas, peak **44** was tentatively identified as hydroxycardadienolide-O-hexoside. It lacks one hydroxyl group compared to digitoxigenin xyloside (peak **40**) and exhibited a protonated ion peak at 519.4 Da. Fragment ion peaks at 357 Da and 339 Da were observed, indicating the loss of a hexose sugar and one water molecule. Additionally, peak **45** was identified as digoxigenin digitoxoside, revealing a protonated ion peak at 521.4 Da. Daughter ion peaks were observed at [M+H-130]^+^ 391 Da, 373 Da, 355 Da and 337 Da, attributed to the loss of a dideoxy hexose sugar moiety and three water molecules (Josephs et al., 2010). Compounds **53** and **57** exhibited fragmentation patterns similar to compound **40**, as they shared the same aglycone structure. Peak **53**, tentatively identified as reevesioside J, exhibited a pseudo-molecular ion peak at 533.4 Da. It showed a daughter peak at [M+H-158]^+^ 375 Da, attributed to the loss of a deoxy methylene hexose (Hsiao et al., 2016). While, peak **57** was identified as ramnodigin, featuring a protonated ion peak at 489.4 Da and showed fragment ions at [M+H-144]^+^ 375 Da attributed to the sequential loss of a trideoxy hexose (Prinsloo, 2007).

Peaks **76**, and **80**-**82** are identified as cardenolide A compounds with sugar moieties doubly linked to the aglycone part. The MS/MS spectra of protonated cardenolide glycosides predominantly exhibit product ions corresponding to the loss of the glycone unit followed by loss of small molecules, namely H_2_O, 2H_2_O, H_2_O + CO and 2H_2_O + CO as observed for peak **80** with a parent ion peak at [M+H]^+^ 531.4 Da and the resulting ions at *m/z* 387, 369, 351, 341 and 323, respectively which was tentatively identified as uscharidin (Cheung & Watson, 1980). The mass difference between [M+H]^+^ ions and daughter ions at *m/z* 387 (aglycone) provided structural information regarding the glycoside units (Kanojiya & Madhusudanan, 2012). Compound **81**, with a parent ion peak at [M-H]^−^ 619.3 Da and fragment ions at 577 Da, 389 Da, and 371 Da, 353 Da, 335 Da resulting from the loss of the acetyl group, sugar moiety and three water molecules, was tentatively identified as acetoxy-hydroxy-norasclepin. While, peak **82** was identified as deoxyuscharin, with a deprotonated ion peak at [M-H]^−^ 572.7 Da and fragment ions at 371 Da, 353 Da, and 335 Da, attributed to the loss of the sugar moiety and two water molecules (Cheung et al., 1983). Finally, peak **76** was tentatively identified as dehydrocalatoxin with a parent ion peak at [M+H]^+^ 547.4 Da and fragment ions at 385 Da, 367 Da, 349 Da, and 339 Da, corresponding to the loss of the sugar moiety, two water molecules, and H_2_O + CO groups (Cheung & Nelson, 1989).

**3.1.6.3. Cardenolides secondary glycosides:**

Secondary cardenolide glycosides were denoted by 11 peaks (peaks **42**, **49**-**51**, **54**-**56**, **58**-**61**). Among these secondary glycosides, coronillobioside (peak **42**) (Gromova et al., 1985) and dihydroxy-oxocardenolide-O-[ hexosyl-deoxyhexoside] (peak **50**) were tentatively identified. Peak **42** displayed a deprotonated ion peak at 711.5 Da, while Peak **50** demonstrated a deprotonated ion peak at 695.4 Da. Both compounds exhibited characteristic daughter ions at 549 Da and 387 Da, indicating the loss of two hexose moieties for peak **42** and the loss of a hexose unit and a deoxy hexose sugar unit for peak **50**. Additionally, both compounds showed daughter ions at 369 Da and 351 Da due to the sequential loss of two water molecules as they share the same oxacardenolide aglycone structure. Peak **49** was identified as dihydroxycardenolide-O-[xylopyranosyl-allopyranoside], showcasing a deprotonated ion peak at 667.5 Da. Fragment ion peaks were observed at 505 Da due to the loss of a hexose sugar, 373 Da due to the loss of a pentose moiety, as well as 355 Da and 337 Da due to sequential loss of two water molecules. The fragmentation pattern observed for compounds **51**, **54**, **56**, **58**, **59** and **61** after losing the sugar moieties were similar to that of compound **49**, sharing the same aglycone part. Peak **51**, tentatively annotated as urezin, exhibited a protonated ion peak at 699.5 Da and ion peaks at 537 Da and 375 Da, attributed to the sequential loss of two hexose units. Peak **54**, identified as dihydroxycardenolide-O-[xylopyranosyl-rhamnopyranoside], exhibited a protonated ion peak at 653.4 Da, with ion peaks observed at 507 Da (loss of a deoxy hexose sugar) and 375 Da (loss of a pentose unit). Similarly, Peak **56**, identified as evobioside, exhibited a protonated ion peak at 683.5 Da. MS^2^ ions were observed at 537 Da, attributed to the loss of a deoxy sugar moiety, followed by an ion peak at 375 Da, resulting from the loss of a hexose moiety (Makarevich, 1972). Moreover, peak **58**, identified as uzarigenin canarobioside, exhibited a protonated ion peak at 667.5 Da, with ion peaks observed at 537 Da (loss of a dideoxy hexose sugar), 375 Da (loss of a hexose sugar) (González et al., 1985). Peak **59**, identified as oxystelmoside, featured a parent ion peak [M+H]^+^ at 667.4 Da. It presented daughter ions at 507 Da, attributed to the loss of deoxy methyl hexose, and at 375 Da, due to the loss of a pentose moiety (Srivastava et al., 1993). Additionally, peak **61** was identified as digitoxigenin bisdigitoxide, featuring a quasi-molecular ion peak [M+H]^+^ at 635.4 Da. Furthermore, it exhibited a daughter ion peak at 505 Da, attributed to the loss of a dideoxy hexose unit, and another at 375 Da resulting from the loss of an additional dideoxy hexose unit. Peaks **55** and **60** also shared the same aglycone structure, resulting in an identical fragmentation pattern after losing the glycone moieties. Peak **55**, identified as digigrandifloroside, displayed a deprotonated ion peak at 681.5 Da, with ion peaks observed at 551 Da and 389 Da (loss of a dideoxy hexose sugar and hexose moieties), as well as fragment ion peaks at 371 Da, 353 Da, and 335 Da (loss of three water molecules) (Kutluay et al., 2019). Peak **60** was tentatively identified as digoxigenin bisdigitoxoside, featuring a protonated ion peak at 651.5 Da. Additionally, it exhibited fragment ions at 521 Da, attributed to the loss of a dideoxy hexose moiety, and at 391 Da, due to the additional loss of another dideoxy hexose moiety. Moreover, the daughter ions observed were similar to those of digigrandifloroside (compound **55**) and were attributed to a three-step dehydration process (K. K. Chen, 1970).

**References:**

Abe, F., Mori, Y., & Yamauchi, T. (1992). Cardenolide glycosides from the seeds of Asclepias curassavica. *Chemical and Pharmaceutical Bulletin*, *40*(11), 2917–2920.

Abe, F., Yamauchi, T., & Wan, A. S. C. (1992). Cardiac glycosides from the leaves of Thevetia neriifolia. *Phytochemistry*, *31*(9), 3189–3193.

Butler, M. S., Towerzey, L., Pham, N. B., Hyde, E., Guymer, G. P., & Quinn, R. J. (2014). Cardenolide Glycosides from Elaeodendron australe var. integrifolium. *Phytochemistry*, *98*, 160–163.

Chen, K. K. (1970). Supplementary glycosides of digitalis. *Journal of Medicinal Chemistry*, *13*(6), 1035–1037.

Cheung, H. T. A., Chiu, F. C. K., Watson, T. R., & Wells, R. J. (1983). Cardenolide glycosides of the Asclepiadaceae. New glycosides from Asclepias fruticosa and the stereochemistry of uscharin, voruscharin and calotoxin. *Journal of the Chemical Society, Perkin Transactions 1*, 2827–2835.

Cheung, H. T. A., & Nelson, C. J. (1989). Cardenolide glycosides with 5, 6-unsaturation from Asclepias vestita. *Journal of the Chemical Society, Perkin Transactions 1*, *9*, 1563–1570.

Cheung, H. T. A., & Watson, T. R. (1980). Stereochemistry of the hexosulose in cardenolide glycosides of the asclepiadaceae. *Journal of the Chemical Society, Perkin Transactions 1*, 2162–2168.

Fabre, N., Rustan, I., de Hoffmann, E., & Quetin-Leclercq, J. (2001). Determination of flavone, flavonol, and flavanone aglycones by negative ion liquid chromatography electrospray ion trap mass spectrometry. *Journal of the American Society for Mass Spectrometry*, *12*(6), 707–715.

González, A. G., Bretón, J. L., Navarro, E., Trujillo, J., Boada, J., & Rodríguez, R. (1985). Phytochemical study of Isoplexis chalcantha. *Planta Medica*, *51*(01), 9–11.

Gromova, A. S., Lutskii, V. I., Semenov, A. A., Valeev, R. B., Kalabin, G. A., & El’kin, Y. N. (1985). Triterpene saponins from Thalictrum minus. V. Structure of thalicoside B. *Chemistry of Natural Compounds*, *21*(5), 629–635.

Heilmann, J., Müller, E., & Merfort, I. (1999). Flavonoid glucosides and dicaffeoylquinic acids from flowerheads of Buphthalmum salicifolium. *Phytochemistry*, *51*(5), 713–718.

Hill, R. A., Makin, H. L. J., Kirk, D. N., & Murphy, G. M. (1991). *Dictionary of steroids*. CRC Press.

Hsiao, P.-Y., Lee, S.-J., Chen, I.-S., Hsu, H.-Y., & Chang, H.-S. (2016). Cytotoxic cardenolides and sesquiterpenoids from the fruits of Reevesia formosana. *Phytochemistry*, *130*, 282–290.

Huq, M. M., Jabbar, A., Rashid, M. A., Hasan, C. M., Ito, C., & Furukawa, H. (1999). Steroids from the Roots of Nerium o leander. *Journal of Natural Products*, *62*(7), 1065–1067.

Hu, Q.-Y., Zhang, X.-K., Wang, J.-N., Chen, H.-X., He, L.-P., Tang, J.-S., Yao, X.-S., & Liu, J. (2021). Malayoside, a cardenolide glycoside extracted from Antiaris toxicaria Lesch, induces apoptosis in human non-small lung cancer cells via MAPK-Nur77 signaling pathway. *Biochemical Pharmacology*, *190*, 114622.

Ibrahim, S. R. M., Mohamed, G. A., Shaala, L. A., Moreno, L., Banuls, Y., Kiss, R., & Youssef, D. T. A. (2014). Proceraside A, a new cardiac glycoside from the root barks of Calotropis procera with in vitro anticancer effects. *Natural Product Research*, *28*(17), 1322–1327.

Josephs, R. D., Daireaux, A., Westwood, S., & Wielgosz, R. I. (2010). Simultaneous determination of various cardiac glycosides by liquid chromatography–hybrid mass spectrometry for the purity assessment of the therapeutic monitored drug digoxin. *Journal of Chromatography A*, *1217*(27), 4535–4543.

Kanojiya, S., & Madhusudanan, K. P. (2012). Rapid identification of calotropagenin glycosides using high‐performance liquid chromatography electrospray ionisation tandem mass spectrometry. *Phytochemical Analysis*, *23*(2), 117–125.

Kutluay, V. M., Makino, T., Inoue, M., & Saracoglu, I. (2019). New knowledge about old drugs; a cardenolide type glycoside with cytotoxic effect and unusual secondary metabolites from Digitalis grandiflora Miller. *Fitoterapia*, *134*, 73–80.

Makarevich, I. F. (1972). Cardiac glycosides of Cheiranthus allioni. VII. *Chemistry of Natural Compounds*, *8*, 191–195.

Marshall, P. G. (1964). Steroids: Cardiotonic glycosides and aglycons; Toad poisons. In *Rodd’s chemistry of carbon compounds* (pp. 360–421). Elsevier.

Parejo, I., Jáuregui, O., Viladomat, F., Bastida, J., & Codina, C. (2004). Characterization of acylated flavonoid‐O‐glycosides and methoxylated flavonoids from Tagetes maxima by liquid chromatography coupled to electrospray ionization tandem mass spectrometry. *Rapid Communications in Mass Spectrometry*, *18*(23), 2801–2810.

Pederson, P. J., Cai, S., Carver, C., Powell, D. R., Risinger, A. L., Grkovic, T., O’Keefe, B. R., Mooberry, S. L., & Cichewicz, R. H. (2020). Triple-negative breast cancer cells exhibit differential sensitivity to cardenolides from Calotropis gigantea. *Journal of Natural Products*, *83*(7), 2269–2280.

Prinsloo, G. (2007). *Isolation of an anti-HIV compound from Elaeodendron croceum (Thunb.) DC.* University of Pretoria.

Qiao, X., Ye, G., Liu, C.-F., Zhang, Z.-X., Tu, Q., Dong, J., Li, Y.-Q., Guo, D.-A., & Ye, M. (2012). Chemical analysis of Eriocaulon buergerianum and adulterating species by high-performance liquid chromatography with diode array detection and electrospray ionization tandem mass spectrometry. *Journal of Pharmaceutical and Biomedical Analysis*, *57*, 133–142.

Ravi, B. G., Guardian, M. G. E., Dickman, R., & Wang, Z. Q. (2020). Profiling and structural analysis of cardenolides in two species of Digitalis using liquid chromatography coupled with high-resolution mass spectrometry. *Journal of Chromatography A*, *1618*, 460903.

Shaker, K. H., Morsy, N., Zinecker, H., Imhoff, J. F., & Schneider, B. (2010). Secondary metabolites from Calotropis procera (Aiton). *Phytochemistry Letters*, *3*(4), 212–216.

Srivastava, S., Khare, M. P., & Khare, A. (1993). Cardenolide diglycosides from Oxystelma esculentum. *Phytochemistry*, *32*(4), 1019–1021.

Zhang, X.-Y., Shen, J., Zhou, Y., Wei, Z.-P., & Gao, J.-M. (2017). Insecticidal Constituents from Buddlej aalbiflora Hemsl. *Natural Product Research*, *31*(12), 1446–1449.


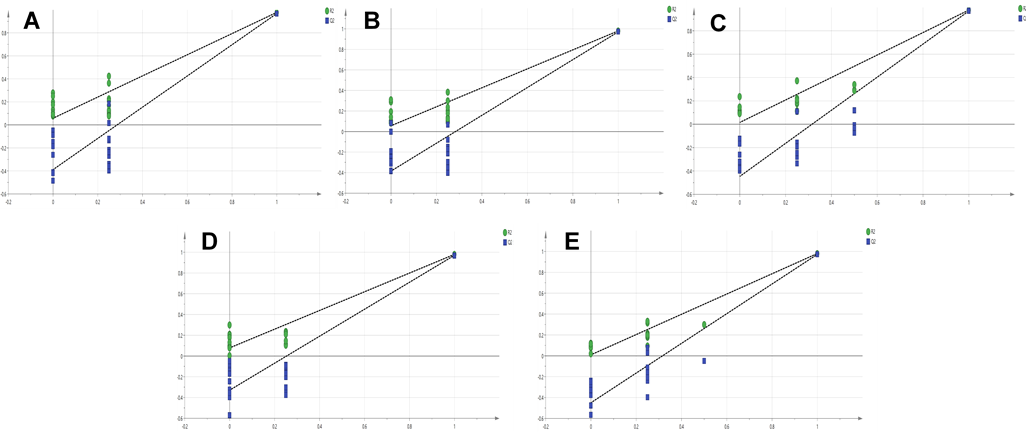


**Figure S1: Permutation plots (20 permutations) of supervised OPLS-DA model of Apple of Sodom samples (A) flowers, (B) fruits, (C) leaves, (D) seeds and (E) stems.**


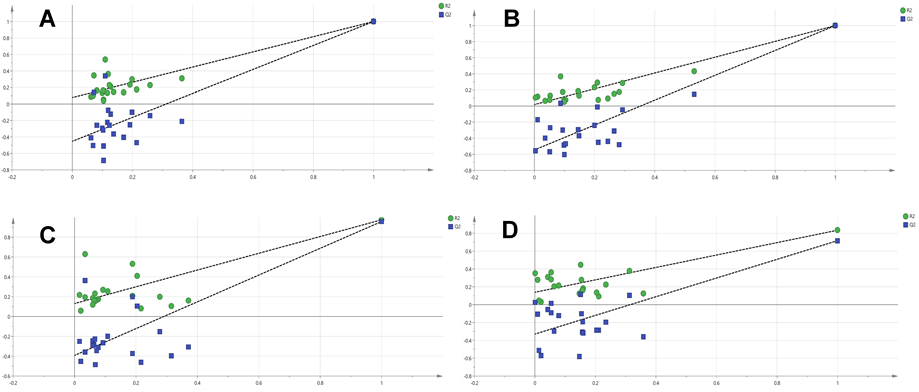


**Figure S2: Permutation plots (20 permutations) of supervised OPLS model on the inflammatory mediators: TNF-*α* (A), IL 1*β* (B), INF-*γ* (C), and IL 6 (D).**
